# Computational evaluation of AKT2 mutations reveals R274H and R467W as potential drivers of protein instability and inhibitor resistance in cancer therapy

**DOI:** 10.1371/journal.pone.0335319

**Published:** 2025-10-27

**Authors:** Sadia Afrin Runa, Mahafujul Islam Quadery Tonmoy, Md. Ashiqul Islam, Md. Aminul Islam

**Affiliations:** 1 Department of Biotechnology and Genetic Engineering, Gopalganj Science and Technology University, Gopalganj, Bangladesh; 2 Department of Biotechnology & Genetic Engineering, Noakhali Science and Technology University, Noakhali, Bangladesh; 3 Computational Biology and Chemistry lab (CBC), Noakhali Science and Technology University, Noakhali, Bangladesh; 4 Department of Biotechnology & Genetic Engineering, Jahangirnagar University, Savar, Dhaka, Bangladesh; 5 Department of Environmental Science & Disaster Management, Gopalganj Science and Technology University, Gopalganj, Bangladesh; King Faisal Specialist Hospital and Research Center, SAUDI ARABIA

## Abstract

Cancer remains a leading cause of mortality worldwide, with genetic alterations such as single nucleotide polymorphisms (SNPs) playing a critical role in tumor progression and therapy resistance. Non-synonymous SNPs (nsSNPs) in AKT2, a key kinase in the PI3K/AKT signaling pathway, can impact protein structure and function, leading to reduced efficacy of targeted cancer therapies. This study employs computational approaches to investigate the structural and functional consequences of nsSNPs in the *AKT2* and their impact on inhibitor interactions. Three structurally and functionally significant nsSNPs (Y265N, R274H, and R467W) were identified where only R274H and R467W were associated with reduced inhibitor binding. R274H, and R467Wwere found to disrupt key molecular mechanisms, including metal binding, loss of allosteric sites, and alterations in post-translational modifications. Molecular docking revealed that R274H, in kinase domain, disrupts key hydrogen bonds with THR292 and GLU279, leading to more flexible binding pocket and significantly reduced binding affinity for Capivasertib and Ipatasertib. Similarly, R467W, in AGC-kinase C-terminal domain, causes the loss of hydrogen bonds with THR292, ASN280, and GLU279, leading to decreased binding affinity for Akt1/Akt2-IN-1, Capivasertib, and Ipatasertib inhibitors. MD simulations further demonstrated that R274H and R467W caused substantial structural deviations and increased residue flexibility, with R467W exhibiting the most pronounced destabilizing effect. These findings suggest that these mutations may contribute to inhibitor resistance by weakening inhibitor interactions and destabilizing the protein-inhibitor complex. This study underscores the importance of genetic screening in optimizing cancer treatment and highlights the need for mutation-specific therapeutic strategies targeting AKT2.

## Introduction

Cancer remains a leading cause of morbidity and mortality worldwide, characterized by uncontrolled cell growth and the ability to invade and metastasize to distant tissues [1]. The underlying mechanisms of cancer development are complex and involve genetic, epigenetic, and environmental factors. Among the genetic alterations implicated in cancer progression, single nucleotide polymorphisms (SNPs) have emerged as critical contributors to tumor initiation, progression, and response to therapy [2]. SNPs, particularly non-synonymous SNPs (nsSNPs), result in amino acid substitutions that can impact protein structure, stability, and function, potentially altering key signaling pathways [3]. The study of nsSNPs has gained prominence in cancer research due to their role in modifying inhibitor responses and contributing to therapy resistance [4].

Recent studies have highlighted the significant influence of nsSNPs on disease susceptibility and therapeutic outcomes. Mutations in key oncogenes and tumor suppressor genes have been associated with variations in inhibitor sensitivity, resistance, and overall treatment efficacy [5]. For instance, mutations in the epidermal growth factor receptor (EGFR) have been linked to resistance against tyrosine kinase inhibitors in non-small-cell lung cancer (NSCLC), whereas BRAF mutations have been shown to drive resistance in melanoma treatment [6]. Additionally, mutations in regulatory proteins such as TP53 and PTEN disrupt normal tumor suppressor functions, leading to uncontrolled proliferation and impaired apoptosis [7]. The presence of such mutations underscores the necessity of personalized medicine approaches that tailor treatment strategies to the genetic profile of individual patients [8].

A growing body of research has established that nsSNPs play a crucial role in modulating the effectiveness of targeted cancer therapies. Structural alterations in proteins caused by nsSNPs can interfere with inhibitor binding, enzymatic activity, and protein-protein interactions [9]. For example, studies have demonstrated that mutations in AKT1, a member of the AKT kinase family, significantly affect inhibitor binding and contribute to therapy resistance [10]. Similarly, alterations in receptor tyrosine kinases and downstream effectors in the PI3K/AKT/mTOR pathway can lead to resistance against inhibitors designed to target these signaling cascades [11–13]. Given the high degree of genetic heterogeneity observed in cancer, comprehensive investigations into the effects of nsSNPs are essential to improving treatment outcomes [14].

AKT2, a key member of the AKT kinase family, plays a fundamental role in regulating metabolism, proliferation, survival, and apoptosis [15]. It is a critical component of the PI3K/AKT signaling pathway, which is involved in insulin signaling, cell growth, and glucose metabolism. AKT2 is predominantly found in insulin-responsive tissues such as the liver, skeletal muscle, and adipose tissue [16]. Genetic alterations have been identified in every level of the PI3K/Akt signalling cascade. The phosphatidylinositol-4,5-bisphosphate 3-kinase, catalytic subunit alpha (PIK3CA), also called p110 alpha protein, is a class I PI3K catalytic subunit that, at first, was considered to be highly involved in ovarian cancers, playing a role as an oncogene [17]*.* Somatic mutations of PIK3CA have also been detected in colorectal, glioblastoma, gastric, breast, lung, and kidney cancers [18–20]. However, somatic mutations in the AKT2 gene, particularly within its kinase domain, have been implicated in cancer development. For example, Parsons *et al.* identified two AKT2 mutations (S302G and R371H) in colorectal cancer, while Soung *et al.* reported a missense mutation (A377V) and two potential splice-site mutations in intron 11 in gastric and lung adenocarcinoma. Given its significant role in oncogenic signaling, genetic variations in AKT, particularly nsSNPs, could have profound implications for cancer treatment.

Despite advancements in targeted therapies, resistance to treatment remains a major challenge in oncology. Inhibitor resistance can arise due to multiple factors, including genetic mutations, epigenetic modifications, and alterations in cellular signaling networks [21]. Inhibitors targeting the PI3K/AKT pathway have shown promise in preclinical and clinical studies; however, the presence of specific nsSNPs in AKT2 may compromise their efficacy [22]. These mutations can lead to conformational changes in the protein structure, affecting ATP-binding affinity and altering the interaction with therapeutic inhibitors. Therefore, understanding the structural and functional impact of nsSNPs in AKT2 is critical for developing more effective and durable therapeutic strategies [23].

This study employs an *in silico* based approach to assess the impact of high-risk nsSNPs in the *AKT2* gene on protein structure and inhibitor interactions. By leveraging molecular docking and MD simulations, we aim to evaluate how these mutations influence AKT2 stability, inhibitor affinity, and overall structural integrity. The findings from this study will contribute to the growing field of precision oncology, emphasizing the importance of genetic screening in optimizing cancer treatment. Understanding the role of nsSNPs in AKT2 can facilitate the development of targeted therapies tailored to individual genetic profiles, ultimately improving patient outcomes and addressing the ongoing challenge of inhibitor resistance in cancer therapy.

## Materials and methods

### Retrieval of relevant dataset

The SNP data (SNP ID, location, variant type, and residue alteration) for the human the *AKT2* gene was retrieved from the National Center for Biotechnology Information (NCBI) dbSNP (Database of Single Nucleotide Polymorphism) database [24] and cross validated using the Ensembl genome browsers’ [25] variant table. The protein sequence (FASTA format) was retrieved from the UniProt database [26] (UniProtKB ID P31751).

### Analysis of the functional consequences of the retrieved SNPs

In the current study, eight web-based bioinformatics tools were utilized to predict the functional impact and pathogenic nature of nsSNPs. All tools were used with their default settings, unless specifically mentioned in the methods.

SIFT (Sorting Intolerant from Tolerant) server [27] was initially used to detect the diseases causing missense SNPs in order to identify potentially deleterious mutations.. SIFT is widely used as a first-pass screening tool for nsSNPs derived from the NCBI dbSNP database, as it allows direct input of batch rsIDs and evaluates the functional impact of amino acid substitutions based on the conservation of homologous sequences [28].. The NCBI rsIDs (accession numbers) of each SNP of the human *AKT2* gene were submitted to SIFT as a predictive question to make prediction. SIFT scores of less than 0.05 were considered deleterious, while those with a score of greater than or equal to 0.05 were tolerated on protein function [27]. The nsSNPs predicted as deleterious by the SIFT server was utilized for further analysis through Polyphen-2 [29], PROVEAN [30], P-MUT [31], PON-P2 [32], Mutation Assessor [33], FATHMM [34], and I-mutant 3.0 [9]. Polyphen-2 uses functional and comparative models produced from sequence information to predict the functional effect of single amino acid substitutions on protein structure. It classifies nsSNPs as possibly damaging (predictive score > 0.15), probably damaging (predictive score > 0.85), or benign (predictive score < 0.15) if amino acids are modified or a mutation is detected in the protein sequence [29]. We submitted the FASTA sequence of AKT2 along with its substitutions and positions in the Polyphen-2 server. The Protein Variation Effect Analyzer (PROVEAN) tool was used to identify amino acid substitution on protein either as deleterious or tolerated. When the probability value for a mutation is less than −2.5 or equal the prediction will be deleterious, otherwise it will be tolerated [30]. We also used the FASTA sequence of AKT2 and amino acid variations as input in the PROVEAN server. P-MUT uses neural networks to predict the pathological character of single amino acid mutation quickly and accurately (with an 80 percent success rate in humans). A predictive score of greater than 0.5 suggests that the nsSNPs are affecting the protein function in a pathological way [31]. UniprotKB id of AKT2 and amino acid variants were submitted as input to the P-MUT server. PON-P2 is another prediction tool that determines whether amino acid substitutions are pathogenic or benign. It’s a machine learning-based tool that takes into account amino acid properties, GENE Ontology (GO) annotations, evolutionarily conserved sequences, and functional annotations for predicting the functional effect of amino acid substitutions on protein structure. The PON-P2 classification system divides variants into three categories: pathogenic, neutral, and unknown [32]. The Mutation Assessor uses the combinatorial entropy optimization and conservation rating to find out the functional residues. The evolutionary conservation of the affected amino acid in protein homologs is used to determine the functional effects of mutations. The production is of two aspects, i.e., FI (functional impact score), and functional (high, medium, neutral) effects [33]. Swiss-Prot id of AKT2 protein and amino acid variants were used as input in the Mutation Assessor server. I-mutant 3.0 is a web server focused on support vector machines (SVMs) that predicts mutation-induced changes in protein stability. I-Mutant analyses the Gibbs free energy (DDG) changes based on the difference between the mutated and native proteins. Neutral mutation (−0.5 ≤ DDG ≥ 0.5 kcal/mol), large decrease (<−0.5 kcal/mol), and large increase (>0.5 kcal/mol) are the three types of I-Mutants 3.0 predictions [9]. In I-mutant 3.0 server, the input was the FASTA file of the AKT2 protein along with the amino acid variations. FATHMM makes use of hidden Markov models (HMMs) to predict the functional effect of protein missense mutations. It assigns a pathogenicity rating representing the average tolerance of the protein/domain to mutations [34]. We used the CScape (Cancer Variant Predictor) module of FATHMM that predicts oncogenic status (disease-driver or neutral) of somatic point mutations within *AKT2* gene sequence. Nucleotide alterations along with their position in the chromosome were used as input in the CScape module of the FATHMM server.

All of the results generated by means of eight algorithms were reviewed, and the nsSNPs that were found to be deleterious in all of the predictions were considered for further analysis.

### Protein evolutionary conservation profile and domain analysis

Consurf [35] was used to predict the conservation profile of amino acids of AKT2 protein. It uses empirical Bayesian inference to estimate the evolutionary conservation of amino acids within a protein sequence. The prediction is categorized into 3 classes: 1–4 score means variable, 5–6 score shows intermediate, and 7–9 rating indicates the conserved amino acid [35]. According to the conservation score and colouring scheme, we predicted the conserved patterns of the amino acids of AKT2. Additionally, structural and functional amino acids were also predicted. For further analysis the pre-selected high-risk nsSNPs located in relatively conserved areas were selected. InterPro tool [37] was used to locate domain regions in AKT2 and to identify the location of nsSNPs in different domains. InterPro is a comprehensive database that provides functional analysis of proteins by classifying them into families and predicting domains and important sites. It integrates data from multiple signature databases, including Pfam, PROSITE, SMART, TIGRFAMs, and CATH-Gene3D to provide a unified view of protein sequence annotations [36].

### Prediction of the molecular effect of amino acid substitutions

All of the prioritized nsSNPs were analyzed through MutPred web server [37] that predicts diseases associated nsSNPs along with the molecular effect of that particular mutation. This offers probabilistic analysis of variants that affect particular aspects of the protein including changes in secondary structure, loss or gain of phosphorylation sites, altered binding sites, disruption of catalytic sites, and changes in solvent accessibility or stability.. Thus, it can help to guide experimental analysis of phenotype-altering variants. MutPred provides a general score, i.e., the likelihood that the amino acid substitution is deleterious, and p-value, i.e., the probability that some functional and structural properties of the protein are impacted [37]. We used the FASTA sequence of AKT2 and amino acid substitutions as inputs. Hypotheses are characterized as a set of values with high g (general score) and low p (probability scores). Actionable hypotheses are described as scores with g > 0.5 and p < 0.05, confident hypotheses are described as scores with g > 0.75 and p < 0.05, and highly confident hypotheses are described as scores with g > 0.75 and p < 0.01 [37].

### Prediction of structural effects upon mutation

HOPE web server [38] was utilized for foreseeing the structural effect of amino acid substitutions on the AKT2 protein. This server used the AKT2 proteins’ tertiary/quaternary structure that are accessible in the Distributed Annotation System (DAS) servers and Uniprot database to identify key structural changes caused by mutations.. Moreover, it provides detailed information on differences between the native and mutant residues, such as alterations in size, charge, and non-covalenthydrophobicity, which may affect the protein’s stability and function [38]. FASTA sequence of AKT2 along with its mutations were taken as input.

### Molecular docking analysis

#### Protein preparation.

The X-ray diffraction crystal structure of the human AKT2 protein was retrieved from the Protein Data Bank (PDB) [39]. We considered the PDB ID: 3D0E for its non-chimeric nature, resolution at a lower wavelength (2.00 Å), and overall quality [Ramachandran outliers (0.2%); Sidechain outliers (0.4%); RSRZ outliers (2.6%); and Clashscore [3]]. Additionally, it contains a well-resolved, co-crystallized AKT inhibitor (GSK690693) with superior structural fit to the experimental electron density, enabling accurate and experimentally supported determination of the active site for docking analysis [40]. Importantly, it has sufficient coverage of the kinase domain including the target mutation sites Y265, R274, and R467. All the chains and hetero atoms in the PDB file were removed except chain A which corresponds to the structure of AKT2 protein. The 3D0E structure contains a mutation at position 474, where serine is replaced by aspartic acid (S474D) [41]. To study the native protein conformation, this mutation was reverted back to the wild-type residue (D474S) using the ‘Mutation tool’ in Swiss PDB Viewer [41]. Energy minimization of the structure was then performed through applying the YASARA force field using the YASARA Energy Minimization Server [42]. Afterwards, the energy minimized protein structure was utilized as template through “Mutation tool” from Swiss PDB viewer for incorporating the identified deleterious nsSNPs (Y265N, R274H, and R467W) into the structure to generate mutant protein structures (each structure with one mutation). Energy minimization of all the generated mutant protein structures was also carried out in the same procedure.

#### Ligand preparation.

Polymorphisms can have an impact on pharmacological responses ranging from potentially lethal adverse inhibitor reactions to equally severe lack of therapeutic effectiveness [43]. In terms of this, an extensive literature review was carried out to find out the inhibitors against AKT2 use in cancer treatment. After review, a number of AKT2 inhibitors were identified (S1 Table). Among them, Capivasertib (FDA approved) and Ipatasertib (phase III trial) were selected for further analysis. Capivasertib, a novel pan-AKT inhibitor, shows significant antitumor activity against hormone receptor-positive advanced breast cancer [44]. In November 2023, the FDA approved the use of Capivasertib with Fulvestrant for patients who have hormone receptor–positive, human epidermal growth factor receptor 2–negative locally advanced or metastatic breast cancer [45]. Ipatasertib, has been extensively tested in phase I and II clinical trials either alone, with chemotherapy, or with hormonal agents. Additionally, phase III trials of Ipatasertib are already under way in HR+ and triple-negative breast cancer [46]. Apart from Capivasertib and Ipatasertib, another small molecule Akt1/Akt2-IN-1 was considered for this study as it is often used in preclinical studies to understand the role of AKT in cellular processes and selective inhibitor resistance. This is due to its specificity for AKT1/2 rather than AKT3 or other members of the AGC kinase family. Akt1/Akt2-IN-1 treatment in an A2780 ovarian carcinoma cell xenograft model showed 80% and 75% inhibition of AKT1 and AKT2, respectively [47]. The 3D chemical structure of the selected inhibitors was extracted from the PubChem database [48] in Spatial Data File (SDF) format and converted to Protein Data Bank (PDB) format by employing PyMOL software [49].

#### Molecular docking.

The impact of the identified deleterious nsSNPs on the therapeutic effectiveness of the selected inhibitors/inhibitors was investigated through molecular docking by implementing AutoDock Vina tool [50]. For this purpose, the wild and mutant AKT2 protein structures were used as receptors and the selected inhibitors (Capivasertib, Ipatasertib, Akt1/Akt2-IN-1) were used as ligands. Prior to docking water molecules were removed from the receptor followed by adding polar hydrogens and united atom Kollman charges to the structure. Gasteiger charge was assigned to the structure of the ligands. To ensure the effective binding of the inhibitors (ligands) in the active site of the protein (receptor), the grid size was determined by Discovery Studio [51] through utilizing a pre-existing AKT2 protein-inhibitor (GSK690693) complex structure (PDB ID: 3D0E). From this, the grid size was designated at dimensions (x, y, and z): 22.320288, −15.739659, and 7.471583 with a grid spacing of 0.375 Å, and the grid center was set to at 40 × 40 × 40 xyz points. The exhaustiveness value was set to 9 to obtain 9 unique conformers for each ligand to balance computational efficiency and conformer diversity. Increasing the exhaustiveness value beyond 9 did not generate additional unique conformers for the ligands used in this study. Finally, AutoDock Vina was employed by utilizing the already prepared protein and ligand structures along with the specified grid box properties in the configuration file. The ligand’s pose with the lowest binding energy was extracted and aligned with the protein structure by PyMOL for further investigation. The post docking analysis was carried out by Discovery Studio and UCSF Chimera [52] to visualize the intermolecular interactions between the protein (native and mutants) and inhibitors/inhibitors.

### Molecular dynamic (MD) simulation analysis

The YASARA Dynamics [53] was used for conducting MD simulation to analyze the dynamic behavior of the protein-inhibitor complexes in a TIP3P water system in different time scales [54] to investigate the binding stability of the inhibitors to proteins (native and mutants). To maintain uniform simulation conditions for each protein-inhibitor complex, a single MD simulation was conducted per system using uniform parameters. The primary step of the simulation was to clean the structure of the protein-inhibitor complexes along with the H-bond network optimization. After that, including a periodic boundary condition a cubic box was generated with a grid size of (96.9654 × 96.9654 × 96.9654) Å. In an explicit water environment to simulate the whole system AMBER14 force field [55] was employed in the periodic cell boundary condition. TIP3P (transferable intermolecular interaction potential 3 points) was implemented for neutralizing the simulation system by the addition of Sodium (Na+) and Chlorine (Cl-) ions. By the steepest descent method, the protein-inhibitor complexes’ energy minimization was carried out. For effective measurement of the interaction of van der Waals and short-range Coulomb, the cut-off radius was restricted to 8 Å. By the PME (Particle Mesh Ewalds) method, the long-range electrostatic interactions were calculated [56]. The whole simulation system was carried out in a particular physiological condition (298 K, pH 7.4, and 0.9% NaCl). For every system, MD simulation was taking place for 100 nanoseconds (ns) with a fixed time step with an interval of 2.5 femtoseconds (fs) at 298 K. At the final stage, evaluation of all the trajectory files were done to obtain root mean square deviation (RMSD), root mean square fluctuation (RMSF), radius of gyration (Rg), solvent-accessible surface area (SASA), and hydrogen bond profile of the simulation system. To check whether each simulation reached a stable and reliable state, key structural properties such as RMSD, RMSF, radius of gyration, and the number of hydrogen bonds were monitored throughout the 100 ns runs. We observed that their values remained relatively stable during the 100 ns simulations, indicating that the systems had equilibrated and were no longer undergoing major structural changes. The MM-PBSA (Molecular Mechanics Poisson-Boltzmann Surface Area) method [57] was utilized to calculate the binding free energy.

## Results

### Retrieval of AKT2 SNPs dataset

**Fig 1:**
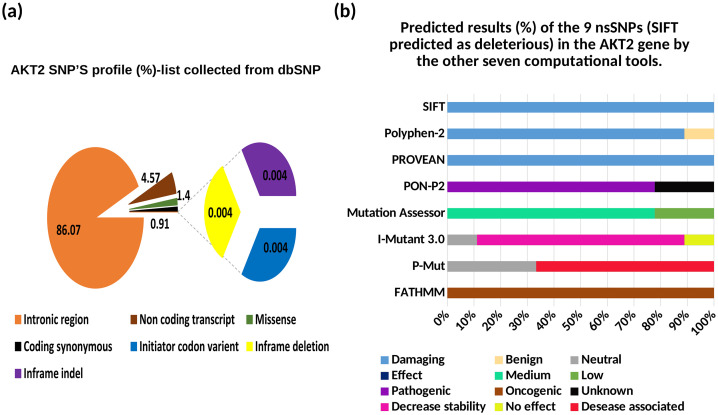
Distribution of SNPs and their frequencies based on mutation classes. **(a)** The pie chart represents the overall distribution of SNPs, highlighting the dominant category and a zoomed-in view of minor categories. **(b)** The bar chart illustrates the percentage of nsSNPs predicted as different functional impacts across eight different algorithms.

The dbSNP database was used to retrieve the full list of *AKT2* SNPs. A total of 21900 SNPs were reported for the human *AKT2* gene which were extracted from the NCBI dbSNP database at the time of our analysis. Out of which, 18850 were found in intron region, 1003 were in non-coding transcript, 308 were missense (nsSNP), 201 were coding synonymous, 1 was initiator codon variant, 1 was inframe deletion and 1 was inframe indel. This information revealed that the maximum number of SNPs were found in the intron region (86.07%), followed by non-coding transcript (4.57%), missense (1.40%), coding synonymous (0.91%), initiator codon variant (0.004%), inframe deletion (0.004%) and inframe indel (0.004%) SNPs, respectively (**Fig 1a**). Among all of those SNPs, only missense SNPs of *AKT2* gene were considered for further analysis.

### Analysis of the functional consequences of the AKT2 nsSNPs

Eight different computational tools were used in our analysis to identify deleterious nsSNPs in *AKT2* (**Fig 1b**). Out of 308 nsSNPs, 9 nsSNPs were predicted by SIFT server to be fundamentally deleterious for protein structure and function. The 25 nsSNPs were predicted to be tolerated by the SIFT server. The remaining 274 nsSNPs were not reported by the SIFT, likely due to insufficient homologous sequence data and their location in non-conserved regions. The 9 deleterious missense SNPs predicted by SIFT were further analyzed for their impact on protein structure and function. For this purpose, we used Polyphen-2, PROVEAN, P-MUT, PON-P2, Mutation Assessor, I-mutant 3.0, and FATHMM. In PolyPhen2, out of 9 nsSNPs, 7 and 1 substitutions were found as probably damaging and possibly damaging, respectively, whereas 1 was identified as benign (neutral). All the nsSNPs predicted as deleterious by the SIFT server were also predicted as deleterious by the PROVEAN server. Out of 9 nsSNPs, 6 nsSNPs were predicted as disease associated mutation by the P-MUT where 3 nsSNPs were recognized as tolerated. PON-P2 identified 7 and 2 nsSNPs as pathogenic variant and unknown, respectively. Out of 9 nsSNPs, 7 mutations having medium impact whereas 2 have low impact on protein structure and function as predicted by Mutation Assessor. Among the 9 nsSNPs, 7 mutations were predicted to be correlated with largely decrease protein stability where 1 mutation has no effect on protein stability and 1 mutation has neutral effect as I-Mutant 3.0 predicted. Finally, we used CScape module of FATHMM to predict the oncogenic status of the selected 9 nsSNPs and all the listed mutations were found to be oncogenic as CScape predicted. All the results are displayed in **Table 1**. All of the results generated by means of eight algorithms were reviewed, and 3 nsSNPs R274H, Y265N, and R467W that were found to be deleterious in all of the predictions were considered for further analysis (**Table 1**).

**Table 1:** Prediction of the effect of the 9 nsSNPs on AKT2 proteins’ structure and function by eight computational tools.

dbSNP	Mutation	SIFT (Score)	P-Mut	Mutation Assessor	Provean	Polyphen2	PON-P	I-Mutant 3.0	FATHMM
rs121434593	R274H	D (0.002)	Disease	M	D	P.D	Pathogenic	Large decrease	Oncogenic
rs387906659	E17K	D (0.008)	Disease	M	D	P.D	Pathogenic	No effect	Oncogenic
rs1804324	Y265N	D (0.003)	Disease	M	D	P.D	Pathogenic	Large decrease	Oncogenic
rs140987550	P314L	D (0.001)	Disease	L	D	P.D	Unknown	Large decrease	Oncogenic
rs142926499	R467W	D (0.008)	Disease	M	D	P.D	Pathogenic	Lerge decrease	Oncogenic
rs144395843	R329W	D (0.007)	Disease	M	D	P.D	Pathogenic	Neutral	Oncogenic
rs200433217	E360A	D (0.044)	Neutral	L	D	P.B	Pathogenic	Large decrease	Oncogenic
rs201145928	P51A	D (0.042)	Neutral	M	D	Po.D	Unknown	Large decrease	Oncogenic
rs374073998	E95K	D (0.017)	Neutral	M	D	P.D	Pathogenic	Large decrease	Oncogenic

Abbreviations: D (deleterious/damaging), M (medium), L (low), P.D (probably damaging), P.B (probably benign), and Po.D (possibly damaging).

### Evolutionary conservation profile and domain analysis of AKT2 protein

**Fig 2:**
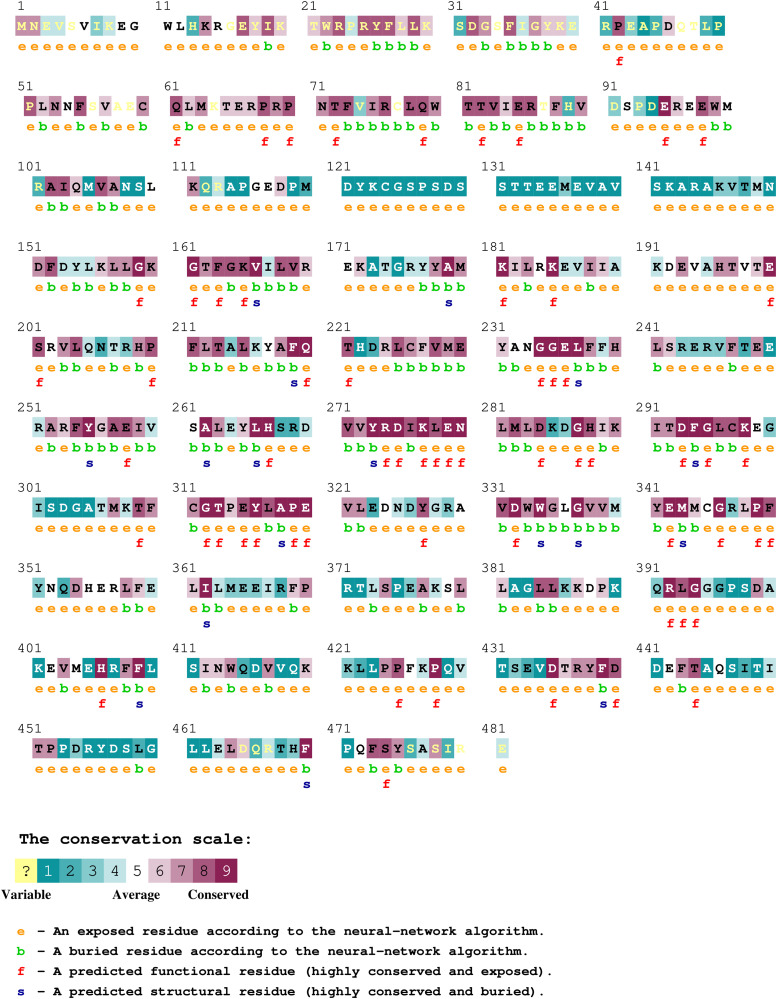
The Fig appears to depict the results of conservation profile of amino acids of protein AKT2.

ConSurf web browser was used to measure the intensity of evolutionary conservation at each residue position in AKT2 protein. By using the Bayesian method, the ConSurf server recognizes putative functional and structural amino acids and identifies their evolutionary conservation profile. The ConSurf analysis predicted that mutant Y265N is buried with a conservation score of 6, mutant R274H is highly conserved and exposed residue with a conservation score of 9 and mutant R467W is exposed residue with a conservation score of 4 (**Fig 2**). InterPro tool was used to locate domain regions in AKT2 and to identify the location of nsSNPs in different domains. It was found that the AKT2 protein contains three distinct domains: the PH domain (amino acids 5–108), the protein kinase domain (amino acids 152–409), and the AGC-kinase C-terminal domain (amino acids 410–481). The nsSNPs (R274H and Y265N) and R467W that were selected are located in the protein kinase domain and AGC-kinase C-terminal domain, respectively (S1 Fig.).

### Prediction of the molecular effect of amino acid substitutions on AKT2 protein

To investigate the pathogenicity of the R274H (Arginine to Histidine), Y265N (Tyrosine to Asparagine), and R467W (Arginine to Tryptophan) nsSNPs and the molecular mechanisms of their pathogenicity, we utilized Mutpred 2 server. The findings showed that the R274H substitution effect the structure and function of the AKT2 protein by altering metal binding, ordered interface, and DNA binding, losing of allosteric site at R274 position and gaining of sulfation at Y273. Similarly, the Y265N mutation effect the AKT2 protein through altering metal binding, ordered interface, and DNA binding, losing of allosteric site at Y265 position and gaining of relative solvent accessibility. The most prominent alteration was observed for the R467W mutation as it impacts the protein by altering disordered and ordered interfece, and transmembrane protein, gaining of pyrrolidone carboxylic acid at Q472, and relative solvent accessibility along with losing O-linked glycosylation at T468, phosphorylation at T468, and ADP-ribosylation at R467. All the findings were statistically significant (P-value < 0.05) and are represented in **Table 2**.

**Table 2:** Predicted pathogenicity of R274H, Y265N, and R467W substitutions and their molecular mechanisms.

rsIDs	Substitution	Effect
rs121434593	R274H	Altered Metal binding (Pr = 0.76 | P = 9.6e-04) Loss of Allosteric site at R274 (Pr = 0.32 | P = 2.6e-03) Altered Ordered interface (Pr = 0.24 | P = 0.03) Altered DNA binding (Pr = 0.17 | P = 0.03) Gain of Sulfation at Y273 (Pr = 0.02 | P = 0.03)
rs1804324	Y265N	Loss of Allosteric site at Y265 (Pr = 0.34 | P = 1.8e-03) Altered Ordered interface (Pr = 0.28 | P = 6.6e-03) Altered Metal binding (Pr = 0.26 | P = 0.01) Gain of Relative solvent accessibility (Pr = 0.25 | P = 0.04) Altered DNA binding (Pr = 0.16 | P = 0.04)
rs142926499	R467W	Altered Disordered interface (Pr = 0.32| P = 0.02) Altered Ordered interface (Pr = 0.29| P = 0.03) Gain of Relative solvent accessibility (Pr = 0.27| P = 0.02) Altered Transmembrane protein (Pr = 0.14| P = 0.02) Gain of Pyrrolidone carboxylic acid at Q472(Pr = 0.05| P = 0.04) Loss of O-linked glycosylation at T468 (Pr = 0.24| P = 0.04) Loss of Phosphorylation at T468 (Pr = 0.34| P = 0.04) Loss of ADP-ribosylation at R467 (Pr = 0.14| P = 0.03)

### Prediction of structural effects of high-risk nsSNPs on AKT2 protein

The structural effects of high-risk candidate nsSNPs on the AKT2 protein from the amino acid substitution was explored by the server Project Hope (S2 Fig.). Substitution (R > H) in 274 positions introduce a smaller netural residue than the positively charged wild-type. The mutation can cause an empty space in the core of the protein structure. The size diference makes that the new H274 residue is not in the correct position to form the hydrogen bond with L296 as the wild-type residue made. The difference in charge might interfere with the ionic interaction (salt bridge) at D332 position formed by the wild-type residue. The mutation introduces an amino acid (H) with different properties which is closely located to the active site and within the protein kinase domain, hence, changing the local structure surrounding the active site and the kinase domain. Substitution of tyrosin by the asparagine in 265 position introduces more hydrophobic smaller residue than the wild-type. The Y265 residue forms a hydrogen bond with Arginine at position 208. The difference in size and hydrophobicity between the wild-type and mutant residue makes that the new Asparagine residue in 265 position is not in the correct position to make the same hydrogen bond along with loss of hydrophobic interactions with other molecules on the surface of the protein. The R467W mutation introduces a more hydrophobic neutral bigger residue than the positively charged wild-type which might lead to structural bumps. The difference in size, charge and hydrophobicity can result in loss of hydrogen bonds and/or disturb correct folding. The mutation is located within AGC-kinase C-terminal domain, therefore can disturb this domain and abolish its function.

### Intermolecular interaction analysis of AKT2 inhibitors with the wild and mutant AKT2 protein

**Fig 3:**
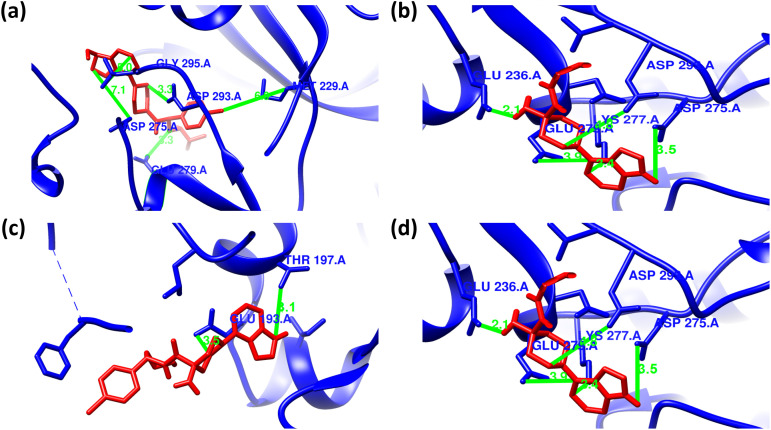
Hydrogen bond interactions between Capivasertib (red) and the AKT2 protein (blue) as predicted by molecular docking analyses. Panels (a–d) represent the docking poses of Capivasertib with wild-type, Y265N, R274H, and R467W forms of AKT2, respectively. **(a)** In the wild-type structure, Capivasertib forms hydrogen bonds with GLY295 (bond length: 3.0 Å), ASP275 (7.1 Å), ASP293 (3.3 Å), GLU279 (3.3 Å), and MET229 (3.6 Å). **(b)** In the Y265N mutant, hydrogen bonding is observed with GLU236 (bond length: 2.1 Å), ASP293 (4.8 Å), LYS277 (3.4 Å), ASP275 (3.4 Å), and GLU279 (3.9 Å). **(c)** For the R274H mutant, hydrogen bonds are formed with THR197 (bond length: 3.1 Å), and GLU193 (3.5 Å). **(d)** In the R467W mutant, Capivasertib forms hydrogen bonds with GLU236 (bond length: 2.1 Å), ASP293 (4.8 Å), LYS277 (3.4 Å), ASP275 (3.4 Å), and GLU279 (3.9 Å).

**Fig 4:**
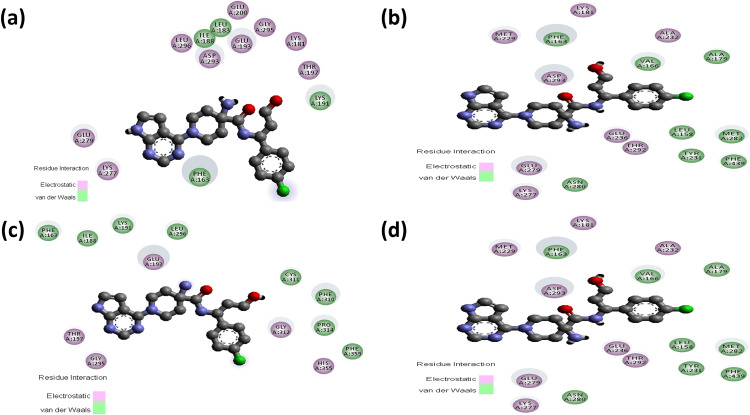
Non-covalent interactions between Capivasertib (ball stick) and the AKT2 protein as predicted by molecular docking analyses. Panels (a–d) represent the docking poses of Capivasertib with wild-type, Y265N, R274H, and R467W forms of AKT2, respectively. **(a)** In the wild-type structure, Capivasertib forms Pi–Pi stacked interaction with PHE163 and a Pi–alkyl interaction with LYS191. **(b)** In the Y265N mutant, Capivasertib interacts through Pi-Stacked bond with PHE163, Pi-alkyl bonds with PHE163, ALA179, and LEU158 alongside several alkyl bonds with MET282, PHE163, ALA179, TYR231, and PHE439. **(c)** In the R274H mutant, Capivasertib engages in a Pi–anion interaction with GLU193 and forms Pi–alkyl and alkyl interactions with PRO314, HIS355, PHE359, ILE188, and LYS191. **(d)** In the R467W mutant, Capivasertib maintains Pi–Pi stacking with PHE163 and forms additional alkyl and Pi–alkyl interactions with ALA179, LEU158, MET282, TYR231, and PHE439.

The consequences of the candidate nsSNPs on the intermolecular interaction of AKT2 with its inhibitor’s molecules. Overall findings showed that the candidate nsSNPs predominantly alter the binding interactions, including binding affinity between the protein and its inhibitors. For Capivasertib, Y265N (−8.7 kcal/mol) gives a sharp increase in binding affinity rather than wild type (−7.7 kcal/mol), where mutants R274H (−6.4 kcal/mol) and R467W (−7.1 kcal/mol), on the other hand, showed decreased binding affinity (**Table 3**). Moreover, Capivasertib provides five hydrogen bonds with GLY295, ASP275, ASP293, GLU279, and MET229 with two non-covalent interactions for wild type protein. Here, Y265N and R467W both provide five hydrogen bonds with two new bonds (GLU236 and LYS277) and lose bonds with GLY295 and MET229, whereas R274H gives two hydrogen bonds where one of them is new (THR197) and simultaneously loses four hydrogen bonds with GLY295, ASP275, GLU279 and MET229 residues. Along with the hydrogen bond, the mutant Y265N and R467W have nine non-covalent interactions and R274H showed seven non-covalent interactions, respectively (**Figs 3 and 4**; S2 Table).

**Table 3:** Binding affinity of Akt1/Akt2-IN-1, Capivasertib, and Ipatasertib with AKT2 (wild and mutants) protein.

Inhibitor	Wild AKT2	Mutant Y265N	Mutant R274H	Mutant R467W
Akt1/Akt2-IN-1	−10.1	−9.9	−10.0	−8.8
Capivasertib	−7.7	−8.7	−6.4	−7.1
Ipatasertib	−7.9	−8.5	−6.6	−6.8

**Fig 5:**
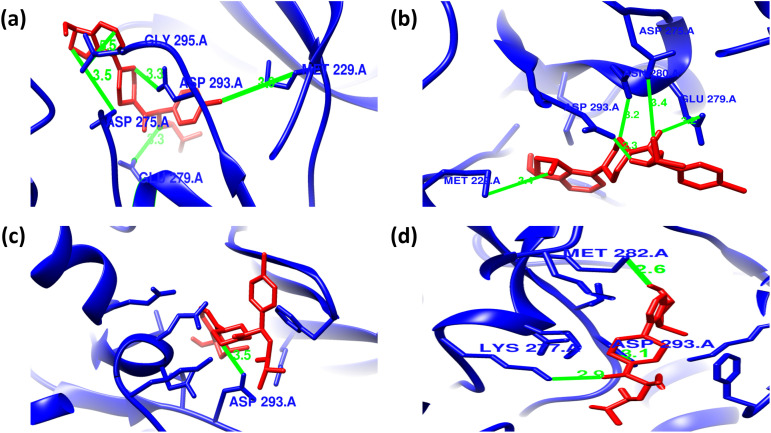
Hydrogen bond interactions between Ipatasertib (red) and the AKT2 protein (blue) as predicted by molecular docking analyses. Panels (a–d) depict the docking of Ipatasertib with wild-type, Y265N, R274H, and R467W forms of AKT2, respectively. **(a)** In the wild-type structure, Ipatasertib forms hydrogen bonds with GLU193 (3.5 Å), ASP275 (3.5 Å), ASP293 (3.3 Å), MET229 (3.8 Å), GLY295 (3.6 Å), and GLU279 (3.3 Å). **(b)** In the Y265N mutant, hydrogen bonds are formed with MET229 (3.4 Å), ASP293 (2.3 Å), GLU279 (3.2 Å), ASN280 (3.2 Å), and ASP275 (3.4 Å). **(c)** In the R274H mutant, Ipatasertib makes only one hydrogen bond with ASP293 (3.5 Å). **(d)** In the R467W mutant, hydrogen bonding involves MET282 (2.6 Å), LYS277 (2.9 Å), and ASP293 (3.1 Å)**..**

**Fig 6:**
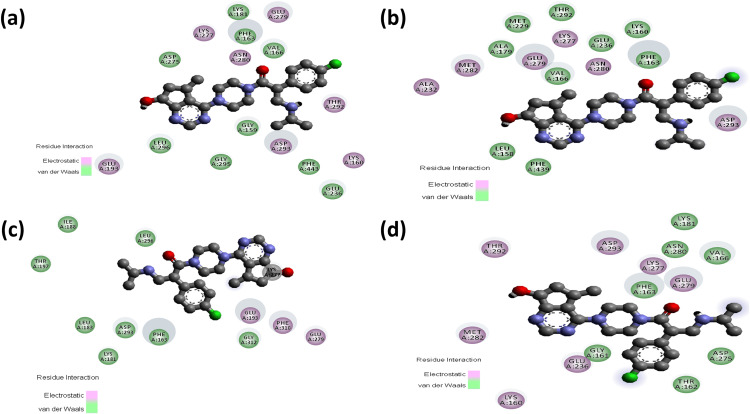
Non-covalent interactions between Ipatasertib (ball stick) and the AKT2 protein as predicted by molecular docking analyses. Panels (a–d) represent the docking poses of Ipatasertib with wild-type, Y265N, R274H, and R467W forms of AKT2, respectively. **(a)** In the wild-type structure, Ipatasertib interacts through Pi–anion bonding with ASP293, and alkyl interactions with MET229. Moreover, Ipatasertib interacts through Pi–alkyl bonding with PHE163, VAL166, and LEU296. **(b)** In the Y265N mutant, additional Pi–sulfur interaction occurs with MET282, Pi–alkyl interactions with LEU158 and alkyl interaction with ILE188. **(c)** For the R274H mutant, non-covalent interactions include alkyl bonds with LEU183 and LEU296. **(d)** In the R467W mutant, Ipatasertib forms Pi–alkyl interactions with PHE163 and VAL166**..**

Ipatasertib has −7.9 kcal/mol binding affinity comparing an increased binding affinity with the mutants Y265N (−8.5 kcal/mol) and lower binding affinity with R274H (−6.6 kcal/mol) and R467W (−6.8 kcal/mol) (**Table 3**). Ipatasertib inhibitor form six hydrogen bond for wild type with GLU193, ASP275, ASP293, MET229, GLY295 and GLU279 including one Pi-Anion bond one Alkyl and three Alkyl bond, after that a gradual hydrogen bond loss was observed with all those nsSNPs such as in R274H that loses five hydrogen bond (GLU193, ASP275, MET229, GLY295 and GLU279) and have only one hydrogen bond, Y265N have five hydrogen bond with one new bond (ASN280) and in R467W, three hydrogen bond visualized among them two of them are new (MET282, LYS277). In Ipatasertib Inhibitor, all the three mutants have lower non-covalent interactions compare to the wild type SNP (**Figs 5 and 6**; S3 Table).

**Fig 7:**
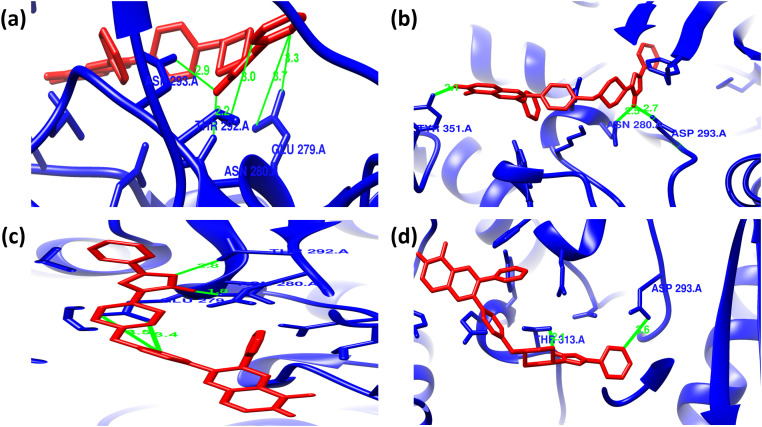
Hydrogen bond interactions between Akt1/Akt2-IN-1 (red) and the AKT2 protein (blue) as predicted by molecular docking analyses. Panels (a–d) represent the docking poses of Akt1/Akt2-IN-1 with wild-type, Y265N, R274H, and R467W forms of AKT2, respectively. **(a)** In the wild-type structure, hydrogen bonds are formed with THR292 (3.0 Å), ASN280 (2.2 Å), ASP293 (2.9 Å), and GLU279 (3.3 Å). **(b)** In the Y265N mutant, Akt1/Akt2-IN-1 forms hydrogen bonds with TYR351 (2.1 Å), ASN280 (2.5 Å), ASP293 (2.7 Å). **(c)** For the R274H mutant, hydrogen bonding occurs with THR292 (2.8 Å), ASN280 (1.8 Å), and GLU279 (3.5 Å). **(d)** In the R467W mutant, hydrogen bonds are seen with THR313 (2.1 Å) and ASP293 (3.6 Å)**..**

**Fig 8:**
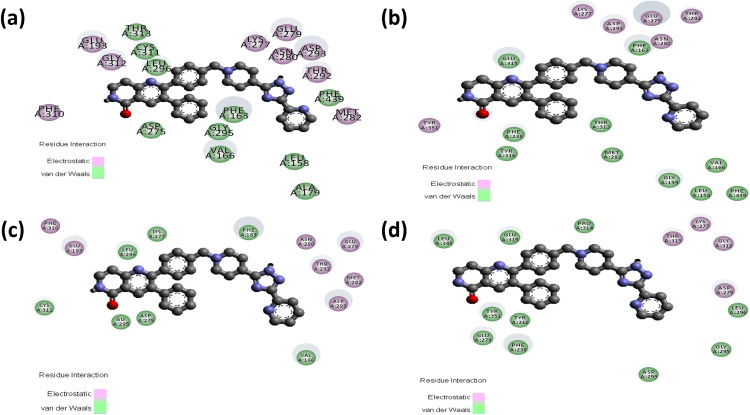
Non-covalent interactions between Akt1/Akt2-IN-1 (ball stick) and the AKT2 protein as predicted by molecular docking analyses. Panels (a–d) show the docking poses of Akt1/Akt2-IN-1 with wild-type, Y265N, R274H, and R467W forms of AKT2, respectively. **(a)** In the wild-type structure, non-covalent interactions include Pi–anion bonding with GLU236, Pi–sigma interactions with LEU296, Pi–sulfur interactions with MET229 and MET282, and Pi–alkyl contact with PHE163 and VAL166. **(b)** In the Y265N mutant, interactions involve Pi–anion bonding with GLU236, Pi-sulfur interactions with LYS277, and Pi-alkyl contacts with GLU279, GLU315, PHE238, PHE163, LYS277, VAL166, and MET282, and Pi-Sigma interaction with GLU315. **(c)** For the R274H mutant, the ligand forms Pi–anion interactions with GLU279 and ASP293, Pi–sigma with LEU296, Pi–sulfur with MET229 and MET282, and Pi–alkyl with PHE163. **(d)** In the R467W mutant, key interactions include Pi–anion bonding with GLU315, Pi–sigma with LEU296 and LEU348, Pi–alkyl with LEU348 and VAL166, and Pi–Pi stacking with PHE238**.**

In case of the inhibitor Akt1/Akt2-IN-1, the mutants Y265N (−9.9 kcal/mol) and R274H (−10.0 kcal/mol) showed similar binding affinity whereas the mutant R467W (−8.8 kcal/mol) lower the binding affinity of AKT2 when compared to wild protein (**Table 3**). Akt1/Akt2-IN-1 inhibitor interacts with the wild AKT2 protein by forming 5 hydrogen bonds with THR292, ASN280, ASP293, and GLU279 residues along with 6 non-covalent interactions. The mutant Y265N forms a new hydrogen bond with TYR351 and simultaneously loses two hydrogen bonds with THR292 and GLU279 residues. Along with the hydrogen bond, the mutant Y265N interacts the inhibitor with 7 and 5 electrostatic and non-covalent interactions, respectively. The mutant R274H showed similar types of intermolecular interactions compared to wild type interactions. Conversely, the mutant R467W forms a new hydrogen bond with THR313 and simultaneously loses three hydrogen bonds with THR292, ASN280 and GLU279 residues along with forming 7 non-covalent interactions (**Figs 7 and 8**; S4 Table).

### Molecular dynamic (MD) simulation analysis of AKT2-inhibitor complexes

In order to determine the geometric and dynamic conformational behavior of the native and mutant protein-inhibitor complexes under a physiological condition, MD simulation was performed at 100 ns timeframe. The structural impact of the candidate mutations (Y265N, R274H, and R467W) on the protein-inhibitor complexes was investigated through this analysis.

#### Structural impact of the candidate mutations on the AKT2- Capivasertib complex.

**Fig 9:**
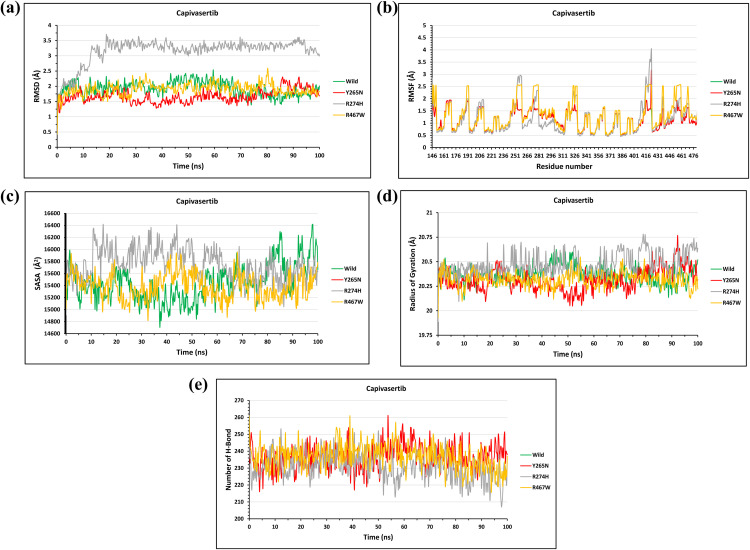
The Fig illustrates the effects of mutations on the stability, flexibility, compactness, and interactions of the AKT2- Capivasertib complex through molecular dynamics simulations. **(a)** RMSD trends showed the wild type remained stable (avg. ~ 1.7 Å), with R274H exhibiting the highest deviation (3.6 Å), and R467W showing abrupt fluctuation after 80 ns. **(b)** RMSF analysis indicated elevated flexibility at residue 431 in R274H, while Y265N and R467W displayed fluctuations comparable to wild type. **(c)** SASA plots showed no major differences in surface exposure across variants, though Y265N displayed minor range expansion. **(d)** Radius of gyration indicates preserved compactness in Y265N, with slight rigidity loss in R274H and R467W. **(e)** Hydrogen bond analysis showed reduced bonding in Y265N and more notably in R274H, whereas R467W retained near-native bonding patterns.

The structural impact of candidate mutations on the AKT2-Capivasertib complex was evaluated using molecular dynamics (MD) simulations. The RMSD analysis provided insights into the stability of the complex over a 100-nanosecond (ns) simulation period. The wild-type AKT2-Capivasertib complex had initiate the effect with an average RMSD of 1.7 Å, showing minor deviations at 30 ns (2.25 Å) and 50 ns (2.5 Å). The Y265N mutation had a minimal effect on the complex, with an initial RMSD of 1.5 Å at 1 ns, which gradually decreased to 1.3 Å at 50 ns, indicating overall stability. In contrast, the R274H mutation introduced significant structural deviations, reaching 3.6 Å at 20 ns, compared to the wild-type’s 2.3 Å at the same time point. The R467W mutation exhibited the greatest instability, with a sudden increase in RMSD at 81 ns (2.6 Å), while the wild-type remained stable at 1.4 Å, suggesting considerable structural disruption (**Fig 9a**).

To evaluate residue-level flexibility, RMSF analysis was performed. The wild-type AKT2 displayed relatively low fluctuations, with an average RMSF of 1.0 Å. However, the Y265N mutation showed minor increases in flexibility, with fluctuations at residue 146 reaching 1.7 Å, compared to 2.6 Å in the wild-type. The R274H mutation displayed the highest flexibility, particularly at residue 431, where fluctuations increased to 4.1 Å, indicating a significant impact on structural dynamics. The R467W mutation showed fluctuations similar to the wild-type, implying no major alterations in flexibility (**Fig 9b**).

To assess the expansion of the protein surface area, SASA analysis was performed. The wild-type AKT2 exhibited SASA values between 14700 Å² and 16450 Å². The Y265N and R467W mutants demonstrated similar fluctuations, with the Y265N mutant reaching highest 15950 Å² at 69 ns and lowest 14800 Å² at 33 ns, whereas the wild-type showed 15940 Å² and 15100 Å², respectively. The R274H mutant showed no significant deviation, suggesting minimal changes in surface accessibility (**Fig 9c**).

The Radius of Gyration (Rg) analysis was conducted to determine the compactness and rigidity of the protein. The Y265N mutant structures exhibited lowest deviation at 51 ns 20.8 Å when the wild type at its highest point that is 20.6 Å additionally at 94 ns the value for this mutant is highest that is 20.78 6 Å when the wild type showed the lowest deviation that is 20.20 Å. The R274H mutant showed slight fluctuations, reaching 20.78 Å at 80 ns, compared to 20.25 Å in the wild-type. The R467W mutant also displayed mild fluctuations, indicating a subtle loss of compactness (**Fig 9d**).

The Hydrogen Bond (H-bond) analysis of the solute (intra-protein and protein-ligand interactions) was conducted to evaluate the internal stability of the AKT2-Capivasertib complex. The wild-type complex maintained stable hydrogen bonding, with a maximum of 260 H-bonds at 40 ns. The Y265N mutant exhibited a slight loss of H-bonds, dropping to 218 H-bonds at 40 ns, compared to 260 H-bonds in the wild-type. The R274H mutant displayed further reductions, with 212 H-bonds at 57 ns, while the wild-type maintained 258 H-bonds. The R467W mutant exhibited mild deviations but retained most hydrogen bonds. These findings suggest that while the Y265N mutation is relatively benign, the R274H and R467W mutations severely compromise the internal structural stability of the AKT2-Capivasertib complex (**Fig 9e**).

#### Structural impact of the candidate mutations on the AKT2-Ipatasertib complex.

**Fig 10:**
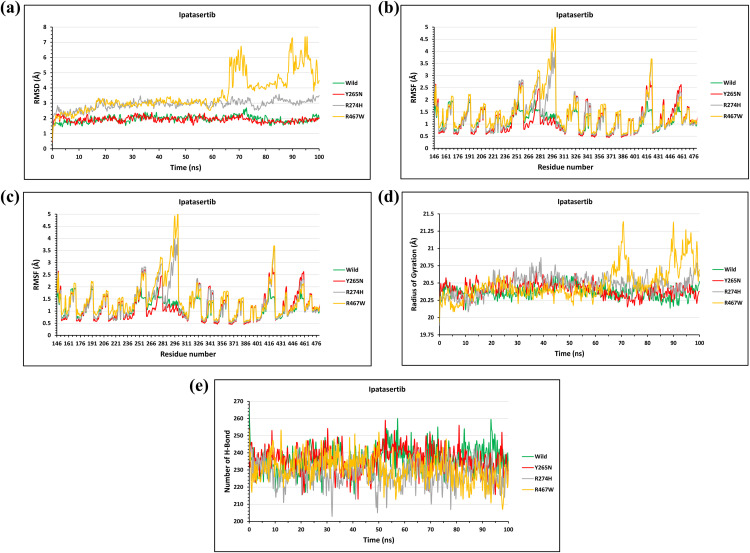
The Fig illustrates the effects of mutations on the stability, flexibility, compactness, and interactions of the AKT2-Ipatasertib complex through molecular dynamics simulations. **(a)** RMSD plots show that Y265N mutation had minimal effect on complex stability, while R274H and R467W caused significant deviations, especially R467W (max 7.3 Å at 95 ns). **(b)** RMSF analysis revealed increased residue flexibility in R274H and R467W mutants compared to native, notably at residues 295 and 296. **(c)** Rg values indicate stable compactness for Y265N, with notable fluctuations in R274H and R467W. **(d)** SASA analysis shows greater surface exposure in R274H and R467W mutants relative to native. **(e)** Hydrogen bond analysis indicates reduced bonding stability in R274H and R467W complexes throughout the simulation.

For the AKT2-Ipatasertib complex, the RMSD was calculated from the Cα atoms of the protein from its initial conformation to determine the stability of the AKT2-Ipatasertib complex. Results showed that the Y265N mutation does not compile much structural deviationas the native and mutant (Y265N) structures maintain their initial conformation till the end where a bit deviation was seen around 2.1Å at 2 ns for the mutant complex while the native complex displayed 1.5 Å. Moreover, at 72 ns the native complex displayed a slight increment in RMSD of around 2.6 Å when compared to the mutant complex showing RMSD of 1.93Å at 72 ns. The average RMSD value of AKT2 (native)-Ipatasertib complex was recorded 1.94Å where AKT2 (Y265N)-Ipatasertib complex showed 1.91Å. The mutant R274H exert a considerable deleterious effect on the AKT2-Ipatasertib complex as a significant structural deviation was observed when compared to native complex. Throughout the simulation period, the AKT2 (R274H)-Ipatasertib complex revealed a noticeable structural deviation with an average RMSD value of 2.9 Å where the average RMSD value of AKT2 (native)-Ipatasertib complex was 1.94Å. The highest deviation was observed for mutant R274H at 85 ns and 97 ns which was 3.5 Å while the AKT2 (native)-Ipatasertib complex showed 2.6 Å at 72 ns. In terms of RMSD, the greatest deviation was seen between AKT2 (R467W)-Ipatasertib complex and AKT2 (native)-Ipatasertib complex. The average RMSD value of AKT2 (native)-Ipatasertib complex was recorded 1.94Å where AKT2 (R467W)-Ipatasertib complex showed 3.5Å. Initially, the value of RMSD was found quite steady but the deviation sudden increase for the AKT2 (R467W)-Ipatasertib complex and it was 6.8 Å at 72 ns. At that time, the AKT2 (native)-Ipatasertib complex showed RMSD value of 2.5 Å. The deviation for AKT2 (R467W)-Ipatasertib complex climbed up the highest 7.3Å at 95 ns where the AKT2 (native)-Ipatasertib complex showed 1.8Å. The results suggested that the AKT2 (R274H and R467W)-Ipatasertib complex are significantly less stable as compared to AKT2 (native)-Ipatasertib complex (**Fig 10a**).

Additionally, RMSF values of mutant and native structures were determined for identifying the mutational effects to dynamic behavior of protein residues. The RMSF value of the residues of native AKT2 and mutant (Y265N) protein was not markedly fluctuated over the entire simulation period. An average RMSF value of 1.0 Å was observed for the native protein whereas the mutant Y265N exhibited 1.1 Å. At the residue 146 the deviation was observed 2.6 Å for Y265N mutant where the native displayed around 1.7 Å. A significant residual fluctuation of 2.5 Å and 2.1 Å was noticed for Y265N mutant at 258 and 281 residues respectfully, whereas the native respectfully showed 2 Å and 1.8 Å. The Y265N mutant showed a fluctuation rate of 2.6 Å and wild type showed 1.9 Å. In terms of RMSF, the mutant R274H showed a considerable deleterious effect on the AKT2 as significant residual fluctuations were observed at multiple sites when compared to native. An average RMSF value of 1.0 Å was observed for the native protein whereas the mutant R274H exhibited 1.4 Å. At the 146, 259, 277, 285, and 289 residues of native AKT2 showed 0.8, 1.1 1.6, 1.1, and 1.7 Å fluctuation rates where mutant R274H displayed 1.8, 2.8, 2.8, 2.0, and 3.1 Å, respectfully. The highest fluctuation took place at 295 residues with a fluctuation rate of 3.9 Å whereas the native showed 1.5 Å Similar to mutant R274H, the mutant R467W showed noticeable residual fluctuation at multiple sites over the entire simulation period. An average RMSF value of 1.0 Å was observed for the native protein whereas the mutant R467W exhibited 1.5 Å. At the 280, 291, and 423 residues of native AKT2 showed 1.3, 1.4, and 1.6 Å fluctuation rates where mutant R467W displayed 3.1, 3.6, and 3.6 Å, respectfully. The highest residual fluctuation took place for mutant R467W at 296 residues of 5Å and at that time the native showed a fluctuation of 1.2 Å (**Fig 10b**).

Furthermore, Rg analysis was carried out for measuring the compactness and rigidity of the AKT2 (native)-inhibitor and AKT2 (mutant)-inhibitor complexes. In terms of compactness and rigidity, the native and mutant (Y265N) structures remained almost close till 100 ns with no significant deviation. An average Rg value of 20.37 Å was observed for the native protein whereas the mutant Y265N exhibited 20.40 Å. For mutant R274H, there was a mild deviation observed as the overall value remains almost steady. An average Rg value of 20.37 Å was observed for the native protein whereas the mutant R274H exhibited 20.47 Å. A prominent deviation of 20.86 Å for mutant R274H was observed at 39 ns where the native showed 20.1 Å. But for mutant (R467W-Ipatasertib) a dramatic fluctuation we can see, at 70 ns wild type display 20.25 Å on the other hand mutant showed 21.40 Å, furthermore we can see at 90 ns mutant (21.35 Å) and wild type (20.15 Å) showed some slight deviation (**Fig 10c**).

Besides, we conducted SASA analysis of the native and mutant complex to determine the expansion of the protein surface area. SASA analysis indicated significant oscillation from mutant (Y265N- Ipatasertib) to the native. The lowest SASA value of 14580 Å2 at 64 ns was observed for mutant protein, whereas native showed 15580 Å2 at 28 ns. Additionally, the highest SASA value for mutant protein was noticed at 45 ns of 15590 Å2, whereas native showed 15090 Å2. For (R274H- Ipatasertib) it was quite steady. At 10 ns wild 15000 Å2 and mutant 16000 Å2 however at 37 ns mutant was 16500 Å2 and wild showed 14680 Å2. Moreover, for mutant (R467W-Ipatasertib) at 30 ns wild type showed 15000 Å2 and for mutant 15500 Å2 and for 70 ns wild showed 15300 Å2 and mutant showed 16500 Å2 (**Fig 10d**).

H-bond analysis demonstrated that the native protein-ligand complex had a maximum number of 260 hydrogen bonds at 54 ns, whereas 259 hydrogen bonds were observed for Y265N- Ipatasertibcomplex. For this complex, the most noticeable hydrogen bond lose was seen between 20–43 ns, but the wild type displayed quite changes of hydrogen bonds over this time. In case of mutant (R274H- Ipatasertib) and (R467W-Ipatasertib) complex there was mild deviation observed. Through the time period wild type complex showed highest value compared to the two mutant protein complex (**Fig 10e**).

#### Structural impact of the candidate mutations on the AKT2-Akt1/Akt2-IN-1 complex.

**Fig 11:**
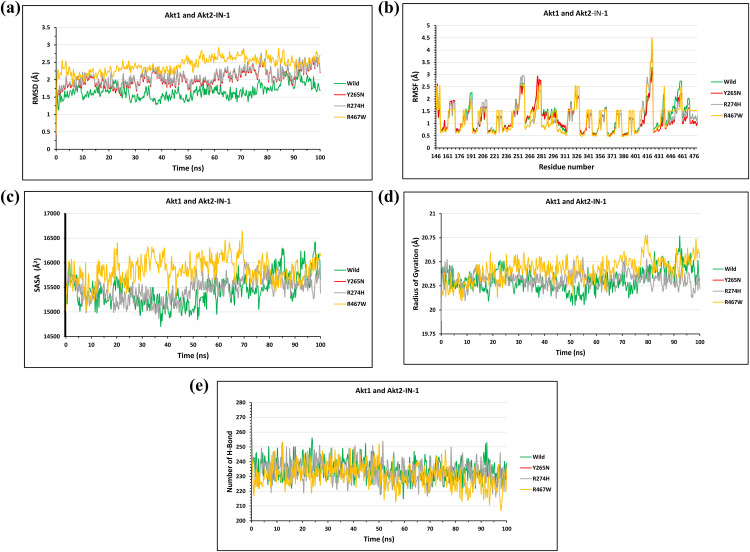
The Fig illustrates the effects of mutations on the stability, flexibility, compactness, and interactions of the AKT2-Akt1/Akt2-IN-1 through molecular dynamics simulations. **(a)** RMSD analysis shows mild deviations for Y265N and R274H mutants, while R467W caused the highest deviation, peaking at 2.9 Å at 64 ns. **(b)** RMSF plots reveal increased residue flexibility in all mutants, with R467W showing the highest fluctuation (4.5 Å at residue 422). **(c)** Rg analysis indicates stable compactness for Y265N and R274H, whereas R467W showed notable structural flexibility. **(d)** SASA results suggest higher surface exposure in all mutants, especially R467W, which reached 16,700 Å² at 70 ns. **(e)** Hydrogen bond analysis revealed reduced bonding in mutants, with R467W showing the most fluctuation.

The stability of the Akt1/Akt2-IN-1 complexes was analyzed by evaluating the deviations of native and mutant structures. From the start of the simulation to its conclusion, the Y265N and R274H mutants exhibited similar deviations. At 10 ns, the wild-type complex maintained a deviation of 1.4 Å, whereas the mutants Y265N and R274H displayed 2.2 Å. By 40 ns, the wild type complex showed a slight increase to 1.3Å, while the mutants Y265N and R274H reached 2.4 Å. At 100 ns, the wild type complex remained at 1.7 Å, whereas the mutants Y265N and R274H structures increased to 2.6 Å. The R467W mutant exhibited the highest RMSD deviation, starting at 2.25 Å at 0 ns, while the wild type showed only 1.5 Å. This deviation continued to increase steadily, peaking at 64 ns R467W reached 2.9 Å, where the wild type measured 1.6 Å (**Fig 11a**).

RMSF analysis revealed inconsistencies in residue fluctuations between the native and mutant structures. The wild type protein displayed relatively stable fluctuations, averaging highest 3.3 Å at residue 416, whereas Y265N and R467W mutants exhibited significant deviations. Specifically, mutant R467W displayed a peak deviation of 4.5 Å at residues 422 whereas the mutant Y265N also show peak deviation of 3.2 Å, and the wild type exhibited 3.2 Å as well. The R274H mutant demonstrated highest fluctuations 3.0 Å at residues 258, while the wild type measured 2.6 Å (**Fig 11b**).

Radius of Gyration (Rg) analysis highlighted variations in protein compactness and rigidity. The Y265N and R274H mutants followed similar compactness trends to the wild type. At 50 ns, the wild type complex maintained an Rg value of 20.15 Å, whereas the mutants Y265N and R274H displayed 20.5 Å. By 93 ns, the wild type complex exhibited 20.75 Å, while the mutants Y265N and R274H remained at 20.25 Å. In contrast, the R467W mutant displayed significant fluctuations, reaching 20.75 Å at 80 ns, while the wild type remained at 20.40 Å (**Fig 11c**).

SASA analysis revealed overlapping fluctuations between Y265N and R274Hin comparison to wild type. At 20 ns, the mutants Y265N and R274HSASA value was 15,000 Å², whereas the wild type measured 15,700 Å². By 73 ns, the mutants Y265N and R274H increased to 16,000 Å², while the wild type decreased to 15,300 Å². The R467W mutant displayed the most distinct variations, with 16,300 Å² at 37 ns compared to 14,600 Å² in wild type. At 70 ns, R467W reached 16,700 Å², while the wild type remained at 15,400 Å² (**Fig 11d**).

H-Bond analysis indicated that the mutant complexes exhibited reduced hydrogen bonding stability. The wild type complex had a maximum of 256 hydrogen bonds at 24 ns, whereas the Y265N and R274H mutant complexescomplexes exhibited similar patterns, maintaining 230 bonds at 24 ns. For both Y265N and R274H mutant complexes, a maximum number of hydrogen bonds 254 was found at 53 ns. The R467W mutant complex displayed significant flactuation, with 253 bonds at 0 ns, whereas the wild type complex retained 242 bonds (**Fig 11e**).

These findings suggest that R467W and R274H mutations significantly compromisethe internal stability of the AKT2-Akt1/Akt2-IN-1 complexes, leading to greater structural deviations and flexibility loss. In contrast, Y265N exhibits a comparatively mild effect on internal stability.

## Discussion

Kinase mutations play a critical role in cancer development and treatment resistance, making their study essential for advancing targeted therapies. The *AKT2* gene, a key component of the PI3K/AKT signaling pathway, is involved in regulating cell proliferation, survival, and metabolism. Mutations within this gene can significantly impact inhibitor efficacy, leading to therapeutic resistance or altered inhibitor interactions. Understanding how specific non-synonymous single nucleotide polymorphisms (nsSNPs) in *AKT2* affect the activity of its inhibitors is crucial for optimizing target specific treatment strategies. This study employs computational approaches to identify and characterize high-risk mutations that may influence the efficacy of AKT2 inhibitors.

A total of 308 nsSNPs were identified in the *AKT2* gene, among which three mutations—R274H, Y265N, and R467W—were consistently predicted to be deleterious across multiple computational tools. These mutations impact protein stability, evolutionary conservation, and functional domains. R274H and Y265N occur within the protein kinase domain, while R467W is located in the AGC-kinase C-terminal domain, both of which are essential for AKT2’s catalytic activity and regulatory functions. These mutations significantly disrupt key molecular mechanisms, including metal binding, loss of allosteric sites, and changes in post-translational modifications such as phosphorylation and glycosylation, potentially altering AKT2’s role in cellular signaling and contributing to cancer progression.

The molecular docking and MD simulations revealed notable differences in inhibitor binding affinities and interaction patterns across the three inhibitors. While Y265N demonstrated improved binding affinity to Capivasertib (−8.7 kcal/mol) and Ipatasertib (−8.5 kcal/mol) compared to the wild-type (−7.7 kcal/mol and −7.9 kcal/mol, respectively), R274H and R467W mutations resulted in decreased binding affinities for all inhibitors. Specifically, R274H (−6.4 kcal/mol for Capivasertib, −6.6 kcal/mol for Ipatasertib, and −10.0 kcal/mol for Akt1/Akt2-IN-1) and R467W (−7.1 kcal/mol for Capivasertib, −6.8 kcal/mol for Ipatasertib, and −8.8 kcal/mol for Akt1/Akt2-IN-1) significantly impaired inhibitor interactions.

Structural analysis indicated that the R274H mutation disrupts key hydrogen bonding and ionic interactions necessary for inhibitor binding, whereas Y265N enhances non-covalent interactions and maintains crucial hydrogen bonds, improving stability for Capivasertib and Ipatasertib. However, R467W introduces steric hindrance and disrupts post-translational modification sites essential for AKT2 function, leading to impaired inhibitor binding across all three inhibitors. Similar patterns have been previously demonstrated in other kinase mutations, such as those in the RET kinase domain, which confer resistance to multiple tyrosine kinase inhibitors including Cabozantinib, Lenvatinib, Vandetanib, and Nintedanib [58].

MD simulations provided additional insights into the stability and flexibility of the mutant protein-inhibitor complexes. The R274H mutation caused significant structural deviations, with an average RMSD of 2.9 Å compared to 1.94 Å for the wild-type. Increased residue flexibility was observed at positions 146, 259, and 289, suggesting that this mutation may reduce the long-term stability of the protein-inhibitor complex. The greater fluctuations in these regions indicate a more flexible binding pocket, which may weaken the inhibitor’s ability to maintain stable interactions with the protein. The R467W mutation had the most pronounced destabilizing effect, with an average RMSD of 3.5 Å and peak deviations reaching 7.3 Å. This mutation exhibited significant flexibility at key residues, particularly 280, 291, and 423, where RMSF values were as high as 3.6 Å. These increased fluctuations suggest that R467W alters the conformational stability of the protein, causing structural disruptions that could lead to inhibitor resistance by weakening inhibitor interactions. The AGC-kinase C-terminal domain, where R467W is located, plays a critical role in AKT2 regulation, and its destabilization may have broader implications on the signaling functions of AKT2 in cancer pathways. The Y265N mutation exhibited relatively stable binding affinity but increased flexibility in specific regions, such as residues 146 and 258. H-bond analysis of the solute also revealed that the internal structure of the Y265N-inhibitor complexes remained relatively stable, allowing to maintain favorable binding conditions. Conversely, R274H and R467W significantly compromise the internal stability of the protein-inhibitor complexes, suggesting unfavorable binding conditions. IHence, overall impact of Y265N on inhibitor binding was less severe compared to R274H and R467W. Overall, the varying effects of these mutations on inhibitor activity have significant implications for AKT2 targeted cancer therapy. When comparing the three mutations across the three inhibitors, Y265N did not negatively affect inhibitor binding, despite being predicted as deleterious based on sequence conservation and loss of native hydrogen bonds. This is likely because newly formed stabilizing hydrogen bonds compensate for the loss, allowing it to maintain its binding ability to Capivasertib and Ipatasertib. This suggests that patients with the Y265N mutation may still benefit from these inhibitors. In contrast, R274H and R467W demonstrated substantial reductions in binding affinity and increased structural destabilization across all three inhibitors. The R467W mutation was particularly detrimental, as it induced significant conformational alterations and reduced inhibitor binding efficiency. Similarly, R274H weakened critical hydrogen bonding interactions, leading to a more flexible binding pocket and reduced inhibitor stability. These observations point to a potential unmet medical need, where patients harboring these deleterious AKT2 mutations may develop resistance to available treatments. The clinical relevance of kinase mutations in mediating drug resistance is well-documented across various cancers. For example, mutations in the BCR-ABL kinase domain have been shown to confer resistance to Imatinib in chronic myeloid leukemia (CML) by disrupting inhibitor binding and reducing therapeutic efficacy [59]. Similarly, JAK2 mutations, particularly V617F, are associated with resistance to JAK inhibitors like Ruxolitinib in myeloproliferative neoplasms [60]. Collectively, these findings emphasize the critical need to consider deleterious mutations into future drug development and clinical strategies to overcome resistance and improve therapeutic outcomes in AKT2-driven cancers.

## Conclusions

This study highlights the critical role of nsSNPs in influencing the efficacy of AKT2 inhibitors, with significant implications for targeted cancer therapy. Using computational approaches, we identified and characterized three high-risk nsSNPs (R274H, Y265N, and R467W) in the *AKT2* gene, which significantly impact protein stability, function, and inhibitor binding affinities, leading to varying effects on therapeutic efficacy. This study highlights the need to consider AKT2 mutations (R274H and R467W) when evaluating targeted therapies, as these mutations reduce binding of AKT2 inhibitors (Capivasertib and Ipatasertib). Consequently, these current AKT2 inhibitors may be less effective for patients carrying these mutations.Future research should focus on validating these computational predictions using multiple MD simulation replicates combined with advanced convergence analysis like PCA, as well as through experimental studies. Additionally, investigating the broader implications of these mutations on AKT2 signaling pathways and their role in cancer progression could provide further insights into personalized therapeutic strategies and might pave the way for more effective and personalized treatment approaches for patients with AKT2 mutations. Ultimately, this study contributes to the growing body of knowledge on kinase mutations and their impact on targeted cancer therapies.

## Supporting information

S1 Fig This Fig provides a comprehensive annotation of a AKT2 protein, depicting its domains, homologous superfamilies, active and binding sites, and other sites. (TIF)

S2 Fig Superimposed structures of AKT2 native (green color) and mutant (red color) models to visualize the stereochemical conformation of wild type and mutant residues at (a) 274, (b) 265, and (c) 467 positions. (TIF)

S1 Table Inhibitors against AKT2 use in cancer treatment. (DOCX)

S2 Table Intermolecular interactions between Capivasertib inhibitor and AKT2 proteins (wild, mutant Y265N, mutant R274H, and mutant R467W). (DOCX)

S3 Table Intermolecular interactions between Ipatasertib inhibitor and AKT2 proteins (wild, mutant Y265N, mutant R274H, and mutant R467W). (DOCX)

S4 Table Intermolecular interactions between Akt1/Akt2-IN-1 inhibitor and AKT2 proteins (wild, mutant Y265N, mutant R274H, and mutant R467W). (DOCX)
