# Supplementary figures and images for: Computational evaluation of AKT2 mutations reveals R274H and R467W as potential drivers of protein instability and inhibitor resistance in cancer therapy
Source: PLoS One. 2025 Oct 27;20(10):e0335319. doi: 10.1371/journal.pone.0335319 (PMC12558497; doi:10.1371/journal.pone.0335319)

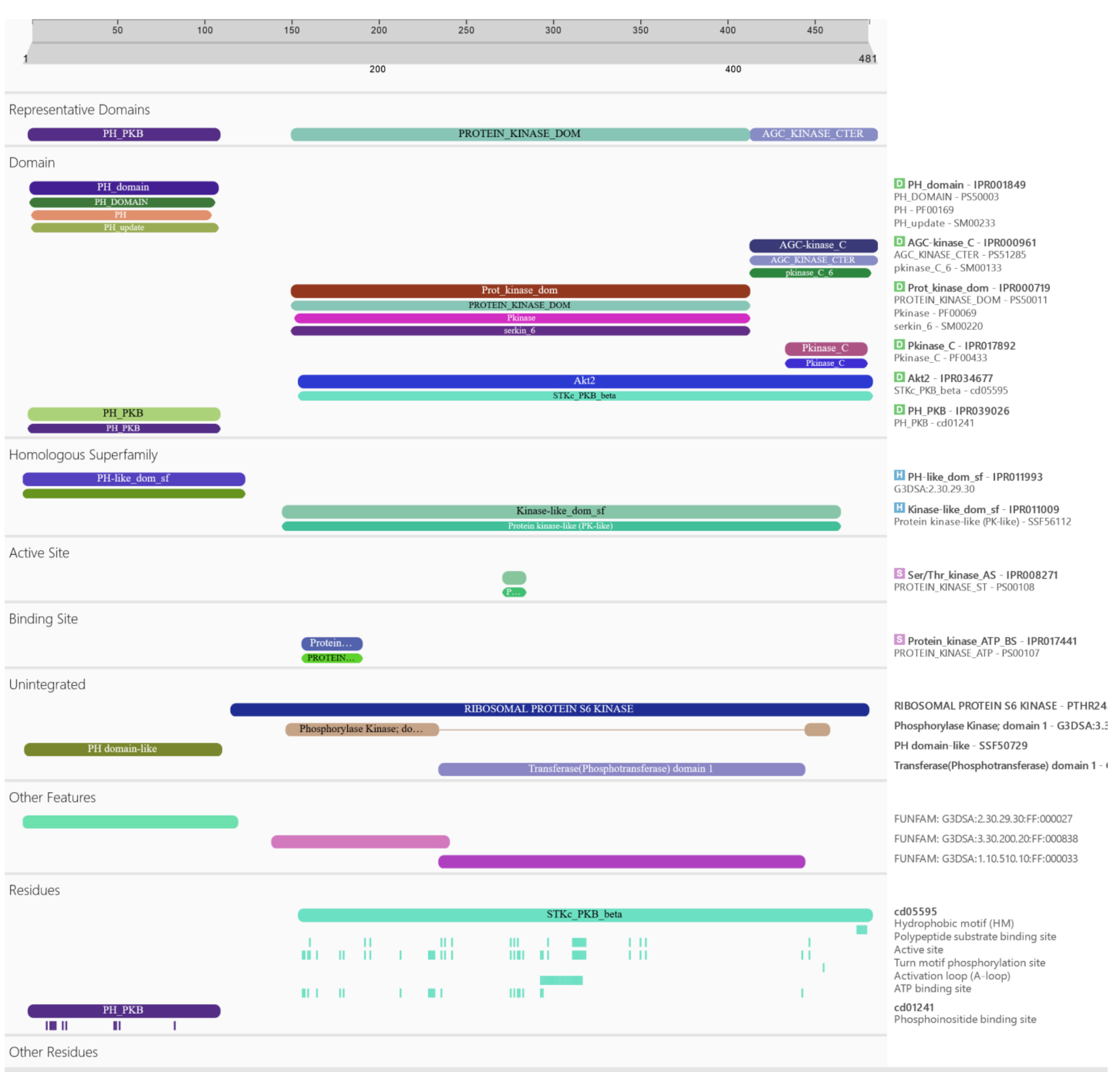

Supplement: S1 Fig — (TIF) [file pone.0335319.s001.tif]

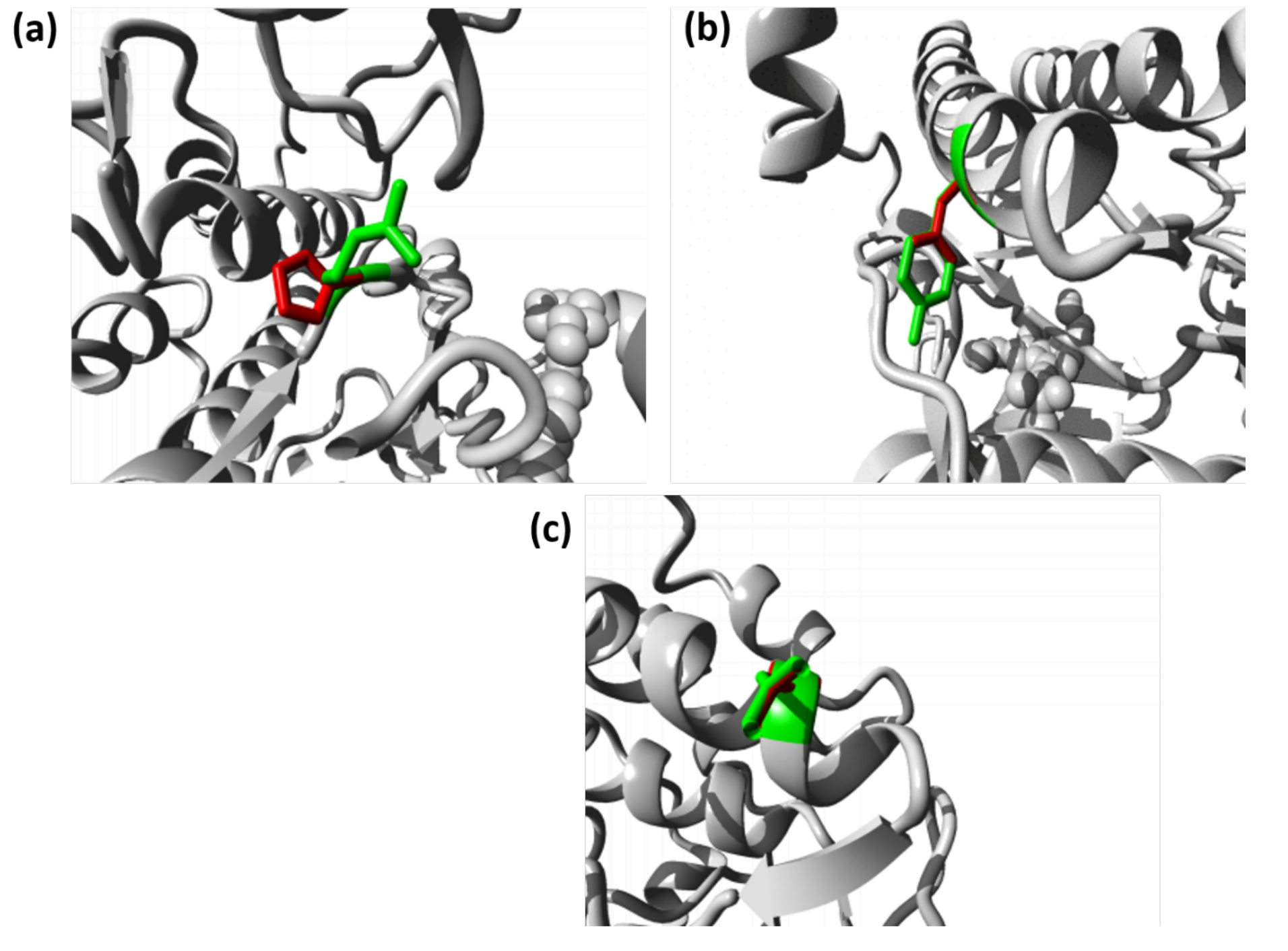

Supplement: S2 Fig — (TIF) [file pone.0335319.s002.tif]
