# Supplementary material for: Computational evaluation of AKT2 mutations reveals R274H and R467W as potential drivers of protein instability and inhibitor resistance in cancer therapy
Source: PLoS One. 2025 Oct 27;20(10):e0335319. doi: 10.1371/journal.pone.0335319 (PMC12558497; doi:10.1371/journal.pone.0335319)
Supplement: S1 Table — (DOCX) [file pone.0335319.s003.docx]

**S1 Table. Inhibitors against AKT2 use in cancer treatment.**

| Si No. | Compound Class | Compound Name | Cancer | Animal Model | Efficacy | Clinical Trial Number | Clinical Phase | Reference |
| --- | --- | --- | --- | --- | --- | --- | --- | --- |
| 1 | Aminofurazan | GSK690693 | Ovarian, prostate, and breast cancer | Mouse | Maximal inhibition of 58 to 75% was observed at the end of dosing period with the 30 mg/kg/day dose | NCT00666081 | Phase I | (1) |
| 2 | Uprosertib | GSK2141795 | Epithelial ovarian cancer | Mice | GSK2141795 reduced FDG signal reaching a maximum of 68% |  | Phase II | (2) |
| 3 | Afuresertib | GSK2110183 | Breast cancer | Mice | No experiment on tumor growth | NCT01532700 | Phase II | (3) |
| 4 | Capivasertib | AZD5363 | Breast and renal cancers | Mice | 100 mg/kg AZD5363 resulted in 80% inhibition (P < 0.0001, HER2þ amplified,PIK3CA-mutant BT474c xenografts) | NCT04305496 | Approved | (4, 5) |
| 5 | Ipatasertib | GDC-0068 | Prostate and ovarian cancer | Mice | 100 mg/kg GDC-0068 qd reached maximum tumor growth inhibition (79%, P < 0.0001); 50 mg/kg GDC-0068 bid resulted in a nearly equivalent tumor growth inhibition | NCT04650581 | Phase III | (6, 7) |
| 6 | Protein Kinase B | MK-2206 | Breast and ovarian cancer | Mouse | MK-2206 alone showed moderate efficacy (P < 0.0001); the combination with lapatinib yielded a significantly greater inhibition of HCC70orSK OV-3 xenograft tumor growth (P < 0.0001) | NCT01333475 | Phase II | (8) |
| 7 | Protein Kinase B | CCT128930 | Glioblastoma and breast cancer | Xenograft (nude) model | 48% and 29% decreases on T/C ratio in U87MG xenografts and BT474 xenografts, respectively |  | Enrolled in clinical trial | (9) |
| 8 | pan-Akt inhibitor | AKT1 and AKT2-IN-1 | Ovarian cancer | Mouse | A statistically significant decrease in mean tumor weight as compared to the vehicle control was noted for mice in the treatment group |  |  | (10) |
| 9 | Inhibitor of AKT1 and AKT2 | BAY 1125976 | Breast and prostate cancer | Xenograft (nude) mouse model | Daily oral treatment with 25 or 50 mg/kg BAY1125976 with T/C volume ratios of 0.14 and 0.08 in KPL-4 xenograft mouse model; with T/C volume values of 0.25 and 0.25 (P < 0.001) in MCF-7xenograftmouse model; with T/C volume values of 0.32 and 0.27 (both P < 0.001) in prostate PDX cancer model | NCT01915576 | Phase I | (11) |
| 10 |  | Perifosine | Gliomas | Mice | 20mmol/L induced cell-cycle arrest at both the G1 and G2–M phases and increased p21WAF1 expression in both tumor suppressor p53 wild-type and knockout cells | NCT00776867 | Phase I | (12, 13) |
| 11 | Natural inhibitors | [6]-Shogaol | Lung cancer | Mouse | Tumor volume decreased 30.2% and 64.2% in the 10 and 40 mg/kg group, respectively; Ki-67 staining showed 56.2% and 93.8% at 10 mg/kg and 40 mg/kg, respectively |  |  | (14) |
| 12 | Natural inhibitors | Oridonin | Esophageal squamous cell carcinoma | Mouse | 160 mg/kg of oridonin significantly reduced tumor growth compared to vehicle group with almost a 50% decrease in Ki-67 staining |  |  | (15) |
| 13 | Natural inhibitors | Herbacetin | Cutaneous squamous cell carcinoma and melanoma | Mouse | Herbacetin treatment significantly decreased then number and volume of skin papillomas relative to the TPA-only-treated group (P < 0.05); it also decreased the volume of melanoma growth relative to the vehicle treated group (P < 0.05) |  |  | (16) |
| 14 | Natural inhibitors | Tehranolide | Breast cancer | Mice | The apoptosis index of the positive group in the tehranolide-treated group was significantly higher than control group (P < 0.01) by TUNEL staining |  |  | (17) |
| 15 | Natural inhibitors | Scutellarin | Hepatocellular carcinoma | Mice | The numbers of lung and intrahepatic metastatic tumors in the scutellarin treated group were significantly less than in the controls (P < 0.05) |  |  | (18) |

1. Rhodes N, Heerding DA, Duckett DR, Eberwein DJ, Knick VB, Lansing TJ, et al. Characterization of an Akt kinase inhibitor with potent pharmacodynamic and antitumor activity. Cancer research. 2008;68(7):2366-74.

2. Cheraghchi-Bashi A, Parker CA, Curry E, Salazar J-F, Gungor H, Saleem A, et al. A putative biomarker signature for clinically effective AKT inhibition: correlation of in vitro, in vivo and clinical data identifies the importance of modulation of the mTORC1 pathway. Oncotarget. 2015;6(39):41736.

3. Dumble M, Crouthamel M-C, Zhang S-Y, Schaber M, Levy D, Robell K, et al. Discovery of novel AKT inhibitors with enhanced anti-tumor effects in combination with the MEK inhibitor. PloS one. 2014;9(6):e100880.

4. Davies BR, Greenwood H, Dudley P, Crafter C, Yu D-H, Zhang J, et al. Preclinical pharmacology of AZD5363, an inhibitor of AKT: pharmacodynamics, antitumor activity, and correlation of monotherapy activity with genetic background. Molecular cancer therapeutics. 2012;11(4):873-87.

5. Turner NC, Oliveira M, Howell SJ, Dalenc F, Cortes J, Gomez Moreno HL, et al. Capivasertib in hormone receptor–positive advanced breast cancer. New England Journal of Medicine. 2023;388(22):2058-70.

6. Blake JF, Xu R, Bencsik JR, Xiao D, Kallan NC, Schlachter S, et al. Discovery and preclinical pharmacology of a selective ATP-competitive Akt inhibitor (GDC-0068) for the treatment of human tumors. Journal of medicinal chemistry. 2012;55(18):8110-27.

7. Martorana F, Motta G, Pavone G, Motta L, Stella S, Vitale SR, et al. AKT inhibitors: new weapons in the fight against breast cancer? Frontiers in pharmacology. 2021;12:662232.

8. Hirai H, Sootome H, Nakatsuru Y, Miyama K, Taguchi S, Tsujioka K, et al. MK-2206, an allosteric Akt inhibitor, enhances antitumor efficacy by standard chemotherapeutic agents or molecular targeted drugs in vitro and in vivo. Molecular cancer therapeutics. 2010;9(7):1956-67.

9. Yap TA, Walton MI, Hunter L-JK, Valenti M, de Haven Brandon A, Eve PD, et al. Preclinical pharmacology, antitumor activity, and development of pharmacodynamic markers for the novel, potent AKT inhibitor CCT128930. Molecular cancer therapeutics. 2011;10(2):360-71.

10. Bilodeau MT, Balitza AE, Hoffman JM, Manley PJ, Barnett SF, Defeo-Jones D, et al. Allosteric inhibitors of Akt1 and Akt2: a naphthyridinone with efficacy in an A2780 tumor xenograft model. Bioorganic & medicinal chemistry letters. 2008;18(11):3178-82.

11. Politz O, Siegel F, Bärfacker L, Bömer U, Hägebarth A, Scott WJ, et al. BAY 1125976, a selective allosteric AKT1/2 inhibitor, exhibits high efficacy on AKT signaling‐dependent tumor growth in mouse models. International journal of cancer. 2017;140(2):449-59.

12. Momota H, Nerio E, Holland EC. Perifosine inhibits multiple signaling pathways in glial progenitors and cooperates with temozolomide to arrest cell proliferation in gliomas in vivo. Cancer research. 2005;65(16):7429-35.

13. Patel V, Lahusen T, Sy T, Sausville EA, Gutkind JS, Senderowicz AM. Perifosine, a novel alkylphospholipid, induces p21WAF1 expression in squamous carcinoma cells through a p53-independent pathway, leading to loss in cyclin-dependent kinase activity and cell cycle arrest. Cancer research. 2002;62(5):1401-9.

14. Kim MO, Lee M-H, Oi N, Kim S-H, Bae KB, Huang Z, et al. [6]-Shogaol inhibits growth and induces apoptosis of non-small cell lung cancer cells by directly regulating Akt1/2. Carcinogenesis. 2014;35(3):683-91.

15. Song M, Liu X, Liu K, Zhao R, Huang H, Shi Y, et al. Targeting AKT with oridonin inhibits growth of esophageal squamous cell carcinoma in vitro and patient-derived xenografts in vivo. Molecular cancer therapeutics. 2018;17(7):1540-53.

16. Kim DJ, Lee M-H, Liu K, Lim DY, Roh E, Chen H, et al. Herbacetin suppresses cutaneous squamous cell carcinoma and melanoma cell growth by targeting AKT and ODC. Carcinogenesis. 2017;38(11):1136-46.

17. Noori S, Hassan ZM. Tehranolide inhibits proliferation of MCF-7 human breast cancer cells by inducing G0/G1 arrest and apoptosis. Free Radical Biology and Medicine. 2012;52(9):1987-99.

18. Ke Y, Bao T, Wu X, Tang H, Wang Y, Ge J, et al. Scutellarin suppresses migration and invasion of human hepatocellular carcinoma by inhibiting the STAT3/Girdin/Akt activity. Biochemical and biophysical research communications. 2017;483(1):509-15.
