# Supplementary material for: Computational evaluation of AKT2 mutations reveals R274H and R467W as potential drivers of protein instability and inhibitor resistance in cancer therapy
Source: PLoS One. 2025 Oct 27;20(10):e0335319. doi: 10.1371/journal.pone.0335319 (PMC12558497; doi:10.1371/journal.pone.0335319)
Supplement: S2 Table — (DOCX) [file pone.0335319.s004.docx]

**S2 Table. Intermolecular interactions between Capivasertib inhibitor and AKT2 proteins (wild, mutant Y265N, mutant R274H, and mutant R467W).**

| **Drug** | **Wild AKT2** | | | **Mutant (265) AKT2** | | | **Mutant (274) AKT2** | | | **Mutant (467) AKT2** | | |
| --- | --- | --- | --- | --- | --- | --- | --- | --- | --- | --- | --- | --- |
| Capivasertib | Interacting residue | Distance | Bond Types | Interacting residue | Distance | Bond Types | Interacting residue | Distance | Bond Types | Interacting residue | Distance | Bond Types |
|  | GLY295 | 3.0 | HB | GLU236 | 2.1 | HB | THR197 | 3.1 | HB | GLU236 | 2.1 | HB |
|  | ASP275 | 7.1 | HB | ASP293 | 4.8 | HB | GLU193 | 3.5 | HB | ASP293 | 4.8 | HB |
|  | ASP293 | 3.3 | HB | LYS277 | 3.4 | HB | GLU193 | 3.6 | Pi-Anion | LYS277 | 3.4 | HB |
|  | GLU279 | 3.3 | HB | ASP275 | 3.4 | HB | PRO314 | 3.9 | Alkyl | ASP275 | 3.4 | HB |
|  | MET229 | 3.6 | HB | GLU279 | 3.9 | HB | HIS355 | 4.2 | Pi-Alkyl | GLU279 | 3.9 | HB |
|  | PHE163 | 5.0 | Pi-Pi Stacked | PHE163 | 5.6 | Pi-Pi Stacked | PHE359 | 4.9 | Pi-Alkyl | PHE163 | 5.7 | Pi-Pi Stacked |
|  | LYS191 | 4.6 | Pi-Alkyl | PHE163 | 4.6 | Pi-Alkyl | PRO314 | 4.9 | Pi-Alkyl | PHE163 | 4.6 | Pi-Pi Stacked |
|  |  |  |  | ALA179 | 3.6 | Pi-Alkyl | ILE188 | 5.0 | Pi-Alkyl | ALA179 | 3.5 | Alkyl |
|  |  |  |  | A:LEU158 | 4.3 | Pi-Alkyl | LYS191 | 5.0 | Pi-Alkyl | LEU158 | 4.4 | Alkyl |
|  |  |  |  | MET282 | 5.1 | Alkyl |  |  |  | MET282 | 5.1 | Alkyl |
|  |  |  |  | PHE163 | 5.3 | Alkyl |  |  |  | PHE163 | 5.2 | Pi-Alkyl |
|  |  |  |  | TYR231 | 4.6 | Alkyl |  |  |  | TYR231 | 4.6 | Pi-Alkyl |
|  |  |  |  | PHE439 | 4.8 | Alkyl |  |  |  | PHE439 | 4.9 | Pi-Alkyl |
|  |  |  |  | ALA179 | 4.6 | Alkyl |  |  |  | ALA179 | 4.6 | Pi-Alkyl |
