# Supplementary material for: Computational evaluation of AKT2 mutations reveals R274H and R467W as potential drivers of protein instability and inhibitor resistance in cancer therapy
Source: PLoS One. 2025 Oct 27;20(10):e0335319. doi: 10.1371/journal.pone.0335319 (PMC12558497; doi:10.1371/journal.pone.0335319)
Supplement: S3 Table — (DOCX) [file pone.0335319.s005.docx]

**S3 Table. Intermolecular interactions between Ipatasertib inhibitor and AKT2 proteins (wild, mutant Y265N, mutant R274H, and mutant R467W).**

| **Drug** | **Wild AKT2** | | | **Mutant (265) AKT2** | | | **Mutant (274) AKT2** | | | **Mutant (467) AKT2** | | |
| --- | --- | --- | --- | --- | --- | --- | --- | --- | --- | --- | --- | --- |
| Ipatasertib | **Interacting residue** | **Distance** | **Bond Types** | **Interacting residue** | **Distance** | **Bond Types** | **Interacting residue** | **Distance** | **Bond Types** | **Interacting residue** | **Distance** | **Bond Types** |
|  | GLU193 | 3.5 | HB | MET229 | 3.4 | HB | ASP293 | 3.5 | HB | MET282 | 2.6 | HB |
|  | ASP275 | 3.5 | HB | ASP293 | 2.3 | HB | PHE163 | 5.0 | Pi-Pi Stacked | LYS277 | 2.9 | HB |
|  | ASP293 | 3.3 | HB | GLU279 | 3.2 | HB | LEU183 | 4.2 | Alkyl | ASP293 | 3.1 | HB |
|  | MET229 | 3.8 | HB | ASN280 | 3.2 | HB | LEU296 | 4.8 | Alkyl | PHE163 | 4.9 | Pi-Alkyl |
|  | GLY295 | 3.6 | HB | ASP275 | 3,4 | HB |  |  |  | VAL166 | 4.8 | Pi-Alkyl |
|  | GLU279 | 3.3 | HB | ILE188 | 4.2 | Alkyl |  |  |  |  |  |  |
|  | ASP293 | 3.3 | Pi-Anion | MET282 | 4.1 | Pi-Sulfur |  |  |  |  |  |  |
|  | MET229 | 5.3 | Alkyl | LEU158 | 5.3 | Pi-Alkyl |  |  |  |  |  |  |
|  | PHE163 | 5.1 | Pi-Alkyl |  |  |  |  |  |  |  |  |  |
|  | VAL166 | 5.4 | Pi-Alkyl |  |  |  |  |  |  |  |  |  |
|  | LEU296 | 4.6 | Pi-Alkyl |  |  |  |  |  |  |  |  |  |
