# Supplementary material for: Computational evaluation of AKT2 mutations reveals R274H and R467W as potential drivers of protein instability and inhibitor resistance in cancer therapy
Source: PLoS One. 2025 Oct 27;20(10):e0335319. doi: 10.1371/journal.pone.0335319 (PMC12558497; doi:10.1371/journal.pone.0335319)
Supplement: S4 Table — (DOCX) [file pone.0335319.s006.docx]

**S4 Table. Intermolecular interactions between Akt1/Akt2-IN-1 inhibitor and AKT2 proteins (wild, mutant Y265N, mutant R274H, and mutant R467W).**

| **Drug** | **Wild AKT2** | | | **Mutant (265) AKT2** | | | **Mutant (274) AKT2** | | | **Mutant (467) AKT2** | | |
| --- | --- | --- | --- | --- | --- | --- | --- | --- | --- | --- | --- | --- |
| Akt1/Akt2-IN-1 | Interacting residue | Distance | Bond Type | Interacting residue | Distance | Bond Type | Interacting residue | Distance | Bond Type | Interacting residue | Distance | Bond Type |
|  | THR292 | 3.0 | HB | TYR351 | 2.1 | HB | THR292 | 2.8 | HB | THR313 | 2.1 | HB |
|  | ASN280 | 2.2 | HB | ASN280 | 2.5 | HB | ASN280 | 1.8 | HB | ASP293 | 3.6 | HB |
|  | ASP293 | 2.9 | HB | ASP293 | 2.7 | HB | GLU279 | 3.4 | HB | GLU315 | 4.2 | Pi-Anion |
|  | GLU279 | 3.3 | HB | LYS277 | 4.8 | Pi-Sulfur | GLU279 | 3.5 | HB | LEU296 | 3.9 | Pi-Sigma |
|  | GLU279 | 3.7 | HB | LYS277 | 4.2 | Pi-Sulfur | GLU236 | 4.9 | Pi-Anion | LEU348 | 3.9 | Pi-Sigma |
|  | GLU236 | 4.9 | Pi-Anion | GLU236 | 4.7 | Pi-Anion | ASP293 | 4.2 | Pi-Anion | PHE238 | 4.1 | Pi-Pi Stacked |
|  | LEU296 | 3.9 | Pi-Sigma | GLU279 | 3.7 | Pi-Alkyl | MET229 | 5.4 | Pi-Sulfur | LEU348 | 5.2 | Pi-Alkyl |
|  | MET229 | 5.5 | Pi-Sulfur | GLU279 | 4.0 | Pi-Alkyl | MET282 | 3.6 | Pi-Sulfur | GLU315 | 4.2 | Pi-Anion |
|  | MET282 | 3.5 | Pi-Sulfur | GLU315 | 4.3 | Pi-Alky | PHE163 | 4.9 | Pi-Alkyl | LEU296 | 3.9 | Pi-Sigma |
|  | PHE163 | 4.9 | Pi-Alkyl | GLU315 | 4.0 | Pi-Sigma | LEU296 | 3.9 | Pi-Alkyl | LEU348 | 3.9 | Pi-Sigma |
|  | VAL166 | 4.1 | Pi-Alkyl | PHE238 | 5.2 | Pi-Alkyl | VAL166 | 4.1 | Pi-Alkyl | PHE238 | 4.1 | Pi-Pi Stacked |
|  |  |  |  | PHE163 | 5.0 | Pi-Alkyl |  |  |  | LEU348 | 5.2 | Pi-Alkyl |
|  |  |  |  | LYS277 | 5.1 | Pi-Alkyl |  |  |  |  |  |  |
|  |  |  |  | VAL166 | 3.9 | Pi-Alkyl |  |  |  |  |  |  |
|  |  |  |  | MET282 | 5.4 | Pi-Alkyl |  |  |  |  |  |  |
|  |  |  |  |  |  |  |  |  |  |  |  |  |
